# Automatic, Qualitative Scoring of the Clock Drawing Test (CDT) Based on U-Net, CNN and Mobile Sensor Data

**DOI:** 10.3390/s21155239

**Published:** 2021-08-03

**Authors:** Ingyu Park, Unjoo Lee

**Affiliations:** Department of Electronic Engineering, Hallym University, Chuncheon 24252, Korea; qkrdlsrb3946@naver.com

**Keywords:** clock drawing test, automatic scoring, wearable sensor, deep learning, U-Net, CNN, MNIST

## Abstract

The Clock Drawing Test (CDT) is a rapid, inexpensive, and popular screening tool for cognitive functions. In spite of its qualitative capabilities in diagnosis of neurological diseases, the assessment of the CDT has depended on quantitative methods as well as manual paper based methods. Furthermore, due to the impact of the advancement of mobile smart devices imbedding several sensors and deep learning algorithms, the necessity of a standardized, qualitative, and automatic scoring system for CDT has been increased. This study presents a mobile phone application, mCDT, for the CDT and suggests a novel, automatic and qualitative scoring method using mobile sensor data and deep learning algorithms: CNN, a convolutional network, U-Net, a convolutional network for biomedical image segmentation, and the MNIST (Modified National Institute of Standards and Technology) database. To obtain DeepC, a trained model for segmenting a contour image from a hand drawn clock image, U-Net was trained with 159 CDT hand-drawn images at 128 × 128 resolution, obtained via mCDT. To construct DeepH, a trained model for segmenting the hands in a clock image, U-Net was trained with the same 159 CDT 128 × 128 resolution images. For obtaining DeepN, a trained model for classifying the digit images from a hand drawn clock image, CNN was trained with the MNIST database. Using DeepC, DeepH and DeepN with the sensor data, parameters of contour (0–3 points), numbers (0–4 points), hands (0–5 points), and the center (0–1 points) were scored for a total of 13 points. From 219 subjects, performance testing was completed with images and sensor data obtained via mCDT. For an objective performance analysis, all the images were scored and crosschecked by two clinical experts in CDT scaling. Performance test analysis derived a sensitivity, specificity, accuracy and precision for the contour parameter of 89.33, 92.68, 89.95 and 98.15%, for the hands parameter of 80.21, 95.93, 89.04 and 93.90%, for the numbers parameter of 83.87, 95.31, 87.21 and 97.74%, and for the center parameter of 98.42, 86.21, 96.80 and 97.91%, respectively. From these results, the mCDT application and its scoring system provide utility in differentiating dementia disease subtypes, being valuable in clinical practice and for studies in the field.

## 1. Introduction

As a sub-test of the Mini-Mental State Examination (MMSE), the Clock Drawing test (CDT) along with the Pentagon Drawing test (PDT) and the Rey–Osterrieth Complex Figure test (ROCF) have been widely used in neuropsychology and related areas for neurological and cognitive exams for dementias such as Alzheimer’s and others [1,2,3,4,5,6,7]. For the CDT, a qualitative neurological drawing test commonly used as a screening instrument for cognitive capabilities, a subject is asked to draw a clock showing a specific time. Placement of the numbers around the clock contour requires visual–spatial, numerical sequencing and planning abilities [8]. Drawing hands indicating a specific time requires long-term attention, memory, auditory processing, motor programming, and frustration tolerance [8]. Hence, in CDT, the test subject uses various cortical areas at the same time for the task being performed; these include use of the frontal, parietal, and temporal lobes [9,10]. As such, various cognitive skills such as selective, sustained attention, visuospatial skills, verbal working memory, visual memory and reconstruction, on-demand motor execution (praxis), auditory comprehension, numerical knowledge, and executive function can be tested [9,11,12]. It was Shulman and collaborators who employed the CDT as a screening tool for older patients for cognitive disorders and, since then, multiple studies have affirmed the utility of the CDT for screening and diagnosis of cognitive impairment for suspected pathologies such as Huntington’s disease, schizophrenia, unilateral neglect, delirium, multiple sclerosis, etc. [13]. Given the utility of CDT, studies on generating a scoring system for CDT are valuable.

To gauge visuo-constructional disorders in moderate and severe dementia, quantitative scoring methods had been developed for the CDT, and these scoring methods have indicated that CDT may be practical in allowing to distinguish the various clinical features of cognitive deficits [14,15,16,17]. However, to improve such methods, neuropsychological approaches using information processing and its qualitative aspects have become necessary, with the aims being to assess the executive functions for the task and analyzing errors in the execution of the drawing. Qualitative approaches analyze various error types such as graphing problems, conceptual deficits, stimulus-bound responses, spatial/planning deficits, and perseveration, and these have helped describe various dementia profiles [18,19]. Furthermore, the assessment of the CDT had been conventionally conducted manually by a medical expert based on identifying abnormalities in the drawings including poor number positioning, omission of numbers, incorrect sequencing, missing clock hands and the presence of irrelevant writing, which is labor intensive, complex and also prone to human subjective errors [20]. Thus, needs of an automatic scoring system have been increased. As such, qualitative and automatic scoring systems are helpful for differential diagnoses [21]. There are several ways of interpreting CDT quantitatively and/or qualitatively [22,23,24,25,26,27,28]. For several of these methods, the scoring involves assessment of drawing parameters, including the size of the clock, the closure of the contour, the circularity of the contour, the existence of the two hands, the proportion of the minute and the hour hands, the positioning of the hands, the correct target numbers of the hands according to the time-setting instruction, the presence of all the digit numbers, the correct position of the digit numbers, the order of drawing of the digit numbers, and the existence of the center in the hand-drawn images for CDT. However, the scoring of CDT is dependent on a clinician’s subjectivity, and so it is prone to be biased and have human errors. Furthermore, it is not very practical in analyzing big data, such as for personal lifelogs. With the CDT results being qualitative and not numeric, it is also difficult to evaluate objectively. Therefore, the necessity of a standardized, qualitative, and automatic scoring system for CDT has been increased. An optoelectronic protocol was suggested to qualitatively induce a parameter related to the movement kinematics in the CDT execution by acquiring the graphic gesture from video recording using six cameras, where a trial duration index was evaluated as a temporal parameter classifying between groups of subjects with Parkinson’s Disease and with both Parkinson’s Disease and dementia [29]. However, the optoelectronic protocol needs a lot of equipment and does not show parametric analysis of the specific behavior patterns during CDT. Due to sensors and sensing systems of modern mobile phones, accurate and rapid measurements of the user’s behavior patterns are available and can be easily implemented without using a lot of hardware equipment. A comparative study was presented for an automatic digit and hand sketch recognition, where several conventional machine learning classification algorithms were considered such as a decision tree, k-nearest neighbors, and multilayer perceptron [30]. Another machine learning based classification was developed for the CDT using a digital pen, where the scoring system used was the original Rouleau scoring system, and a comparative study was executed on the digital pen stroke analysis [31]. A deep learning approach was recently applied for automatic dementia screening and scoring on CDT, where the screening was for distinguishing between sick and healthy groups without scaling on the levels of dementia, and the scoring was for a performed dementia test not for an assessment of CDT [8]. In other words, the deep learning approach scored the CDT image in six levels according to the severity and status of dementia by using the image classification neural networks, such as VGG16 [32], ResNet-152 [33], and DenseNet-121 [34]. Therefore, a qualitative and automatic scoring system for CDT in mobile environments is still strongly demanded.

For this study, a mobile-phone app version for CDT, namely mCDT, was developed that includes a novel, original, automatic, and qualitative scoring system. The scoring methodology employs U-Net, a convolutional network for biomedical image segmentation, CNN, a convolutional network for digit classification, and MNIST database, the Modified National Institute of Standards and Technology database. Smart phone mobile sensor data were used to develop mCDT and its scoring system. Hand drawn clock images numbered 159 and were of 128 × 128 resolution; they were obtained via mCDT to train the U-Net to generate the trained model, DeepC, tasked with segmenting the contour of a drawn clock image. Similarly, the U-Net was trained with the same 159 CDT 128 × 128 resolution images to obtain DeepH, also a trained model, designed for segmenting the hands of a drawn clock image. The MNIST database was used to train CNN to obtain the trained model, DeepN, employed for classifying the digit images from a hand drawn clock image. The accuracies of greater than 75% and those being saturated were obtained with the epochs iterated for DeepC and DeepH. Similarly, accuracies of greater than 98% that were saturated were achieved with epochs iterated for DeepN. The mobile sensor data were the x and y coordinates, timestamps, and touch events for all the samples with a 20 ms sampling period extracted from the mobile touch sensor. Four parameters including contour (0–3 points), numbers (0–4 points), hands (0–5 points), and the center (0–1 points) were estimated by using DeepC, DeepH and DeepN along with the sensor data, resulting in scaling with a total of 13 points.

As a result, this study comes up with a significantly effective and accurate CDT scoring system available in a mobile device, which not only achieves the state-of-the-art accuracy on the assessment and the scaling of each CDT parametric criterion, but is also applicable to neurological disease diagnosis as well as temporal difference assessment of cognitive functioning in a daily lifelog. Especially, it is noticeable from the performance results for this system to show relatively more excellent specificity and precision for the PD test group than the young volunteer group. These results suggest that our mCDT application and the scoring system are valuable in differentiating dementia disease subtypes and also useful for clinical practice and field studies.

The rest of this paper is organized as follows. Section 2 describes the number of subjects enrolled in this study and the information related to the obtained approval of the ethics committee for gathering data from the subjects and the protocols suggested in this study. Section 2 also describes the implementation of mCDT, a mobile-phone app of CDT, the training models DeepC, DeepH and DeepN generated in this study, and the CDT scoring methods using the training models and the sensor data collected from mCDT for each of the parameters, such as the contour, numbers, hands, and center. Section 3 describes the results of the performance test of mCDT on a case-by-case basis for each of the four parameters. Section 4 presents a discussion of the results, the limitations, and the future works. Finally, Section 5 summarizes the overall results of this study.

## 2. Materials and Methods

### 2.1. Subjects

Right-handed young volunteers (238 total, 147 males and 89 females, aged 23.98 ± 2.83 years) were enrolled and took part in developing mCDT, the deep learning based mobile application in this study. Parkinson’s disease (PD) patients visiting the university hospital were also part of the study, submitting clock drawing images (140 total, 76 males and 64 females, aged 75.09 ± 8.57 years). The above-mentioned 238 volunteers were used to create the pre-training models of DeepC and DeepH. Image data from 159 volunteers (112 males and 45 females, aged 24.78 ± 1.63 years) and their clock drawing were used for the U-Net algorithm. The remaining 79 volunteers (35 males and 44 females, aged 22.81 ± 0.79 years) and those from the 140 PD patients with their clock drawing image data were subjected to testing the application’s scoring method. Two neurologists assisted in scoring of the CDT as the experts in CDT scaling, and cross checked all the images for an objective performance analysis, as well as gathering the data of the CDT test from the 140 PD patients. The Institutional Review Board of the Hallym University Sacred Heart Hospital, as an independent ethics committee (IEC), ethical review board (ERB), or research ethics board (REB), approved the gathering data and the protocols used for this study (IRB number: 2019-03-001). Table 1 provides the age, gender, and binary CDT score summary of the 238 volunteers and 140 PD patients.

### 2.2. Implementation of the Deep Learning Based Mobile Clock Drawing Test, mCDT

**Figure 1:**
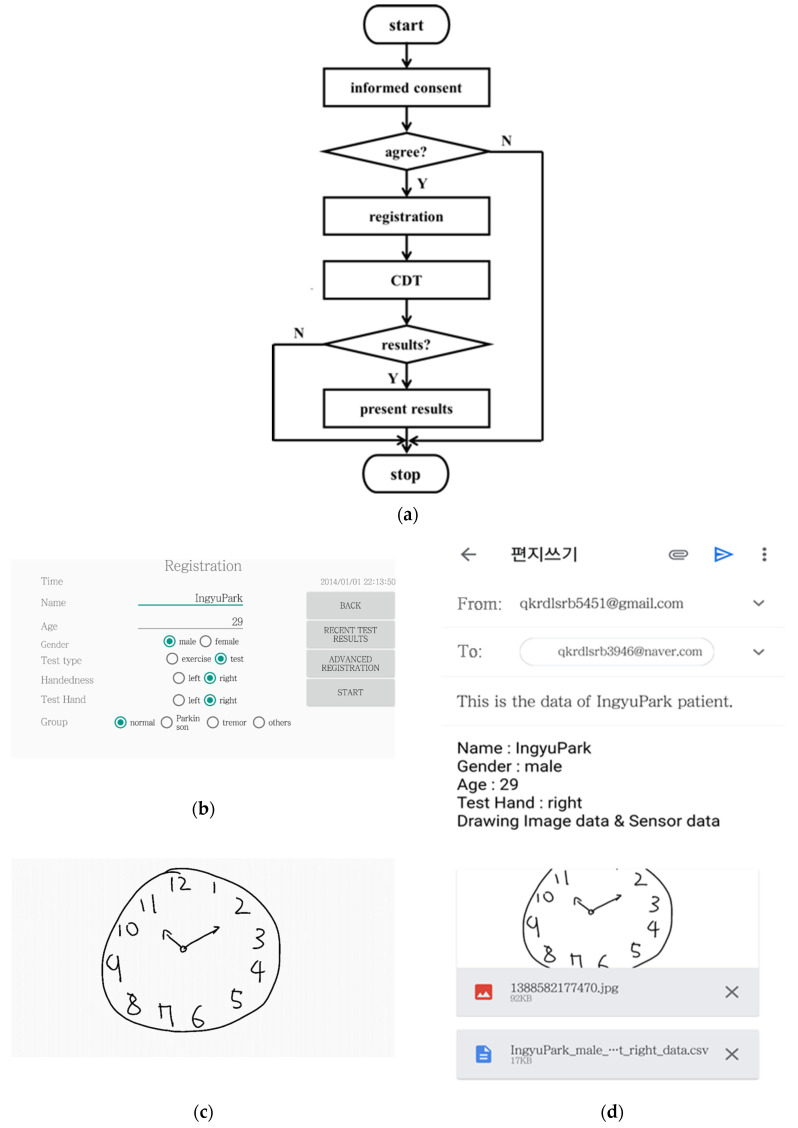
(**a**) Flow diagram of mCDT operation; screen shots of (**b**) registration window; (**c**) CDT window; and (**d**) results window of mCDT.

The Android Studio development environment was used to develop the deep learning based mobile application mCDT for the clock drawing test. A user of mCDT draws the face of a clock with all the numbers present and sets the hands to a specific time such as 10 after 11. Here, the clock face contour could be pre-drawn by mCDT as an option chosen by the user and the specific time is randomly selected by mCDT. Then, mCDT scores the drawn image qualitatively; this scoring is based on mobile sensor data of the drawing image and pre-trained models, DeepC, DeepH and DeepN created in this study. Fast and precise segmenting of the clock face contour and the hands in the images were accomplished by DeepC and DeepH, respectively, using U-Net, a convolutional network architecture. In turn, DeepN classifies the numbers using CNN and the MNIST database. The mobile sensor data of x and y coordinates in pixels, timestamps in seconds, and touch events for each samples of the drawing image are made with a 50 Hz sampling frequency. Three types of the touch events, ‘up’, ‘down’, and ‘move’ were considered in mCDT. The touch event ‘down’ occurs when the user starts to touch on the screen; ‘up’ when the user ends it; and ‘move’ when the user moves the finger or the pen across the screen. Figure 1a provides the flow chart of the processes by mCDT. Figure 1b–d provide the screen shots of the registration window, the CDT window, and the result window of mCDT, respectively. As shown in Figure 1a,b, an informed consent prompt appears at the launch of mCDT, followed by a registration window for entering the subject’s information; these include age, name, gender, education level and handedness of the subject plus optional parameters including an email address. After pressing the start button in the registration window, the CDT window appears as shown in Figure 1c, and the user is instructed to draw numbers and hands on a clock face contour; the contour of the clock face is asked to be drawn by the user or pre-drawn by mCDT as an option chosen by the user. In the drawing, the clock hands have to set to a specific time given randomly by mCDT. The sensor data are saved as the subject draws a contour, numbers and hands of a clock face on the touch screen of the CDT window. The sensor data along with the drawn image are then provided in the results window as shown in Figure 1d. The results could then be forwarded to the email address submitted at the registration window.

### 2.3. Pre-Trained Models, DeepC, DeepH and DeepN Based on the U-Net and the CNN

**Figure 2:**
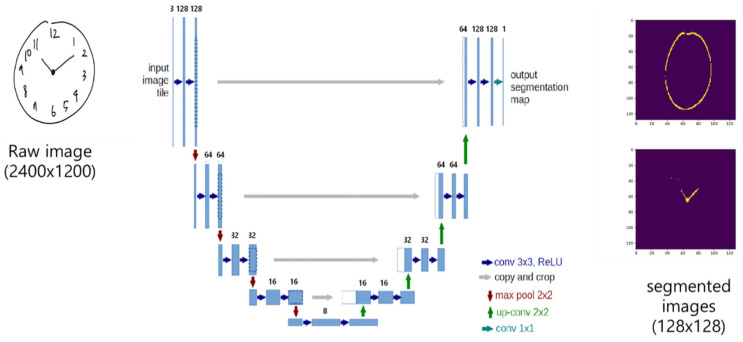
U-Net network architecture for this study. The U-Net network architecture is composed of a contracting path, an expansive path, and a final layer. The contracting path is made up of repetitive applications of two 3 × 3 convolutions and a 2 × 2 max pooling operation with stride 2 for down-sampling. The expansive path exists of two 3 × 3 convolutions and a 2 × 2 convolution for up-sampling. The final layer employs a 1 × 1 convolution for mapping each 16-component feature vector for the required number of classes.

**Figure 3:**
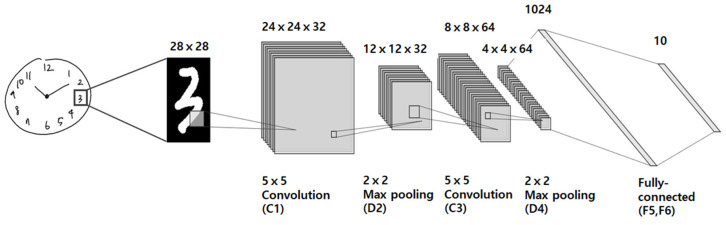
CNN network architecture for this study. It is made up of two convolution layers (C1 and C3), two pooling layers (D2 and D4), and two fully connected layers (F5 and F6). C1, the first convolution layer, filters the 28 × 28 input number image with 32 kernels of 5 × 5 size; C3, the second convolution layer, filters the down-sampled 12 × 12 × 32 feature maps with 64 kernels of 5 × 5 × 32 size. Both of the convolution layers use a unit stride, and at the output of each, a ReLU nonlinear function is used. At layers D2 and D4, down-sampling is performed with 2 × 2 non-overlapping max pooling. For the two final fully-connected layers, F5 and F6, they respectively have 1024 and 10 neurons.

Novel pre-trained models DeepC, DeepH and DeepN were developed for the segmentation and classification of the contour, the hands, and the numbers, respectively, of the clock face from a drawn clock image. DeepC and DeepH were created based on the U-Net convolutional network architecture and DeepN, based on the CNN in keras [6]. The U-Net and CNN network architecture implemented in this study are illustrated in Figure 2 and Figure 3, respectively. The U-Net network architecture consists of a contracting path, an expansive path, and a final layer as shown in Figure 2. The contracting path consists of repeated applications of two 3 × 3 convolutions and a 2 × 2 max pooling operation with stride 2 for down-sampling. At each repetition, the number of feature channels is doubled. The expansive path consists of two 3 × 3 convolutions and a 2 × 2 convolution (“up-convolution”) for up-sampling to recover the size of the segmentation map. At the final layer, a 1 × 1 convolution was used to map each 16-component feature vector to the desired number of classes. In total, the network has 23 convolutional layers. The training data for both DeepC and DeepH contain 477 images of 128 × 128 resolution, which were augmented using a module called ImageDataGenerator in keras.preprocessing.image and resized from the original 159 images of 2400 × 1200 resolution. The augmentation was carried out by randomly translating horizontally or vertically using the parameter value 0.2 for both width_shifting_range and height_shifting_range. DeepC and DeepH were both trained for 100 epochs with an accuracy of about 77.47% and 79.56%, respectively. The loss function used for the training was basically a binary cross entropy. The CNN network architecture consists of two convolution layers (C1 and C3), two pooling layers (D2 and D4), and two fully connected layers (F5 and F6), as shown in Figure 3. The first convolution layer C1 filters the 28 × 28 input number image with 32 kernels of size 5 × 5, while the second convolution layer C3 filters the down-sampled 12 × 12 × 32 feature maps with 64 kernels of size 5 × 5 × 32. A unit stride is used in both the convolution layers, and a ReLU nonlinear function is used at the output of each of them. Down-sampling occurs at layer D2 and D4 by applying 2 × 2 non-overlapping max pooling. Finally, the two fully-connected layers, F5 and F6, have 1024 and 10 neurons, respectively. The MNIST handwritten digit database (about 60,000 images) and the digit images from the 477 CDT images were used to train the CNN architecture used here to obtain the trained model, DeepN.

### 2.4. Scoring Method of mCDT

**Figure 4:**
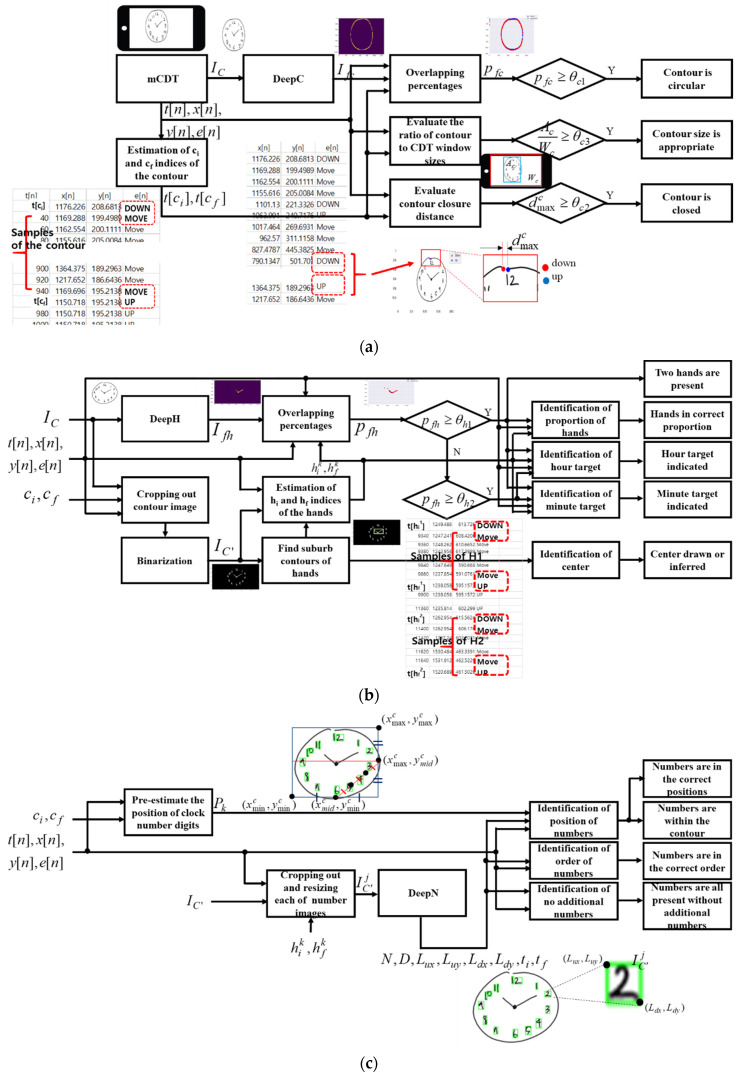
Overall flowchart of the scoring method, (**a**) for parameter contour, (**b**) for parameters hands and center and (**c**) for parameter numbers.

The novel, automatic and qualitative scoring method of mCDT was developed, based on the sensor data and the pre-trained models, DeepC, DeepH and DeepN. Four parameters were included in the scoring method: contour (0–3 points), numbers (0–4 points), hands (0–5 points), and the center (0–1 point). Some of the scoring criteria were adopted from a previous study by Paolo Caffarra et al. [22]. A total score corresponding to the sum of individual scores of each parameter ranged from 0 to 13. When a subject executes the CDT more than once, the best copy is then scored. A detailed list of the scoring criteria of each parameter used in this study is presented in Table 2 and the overall flowchart of the scoring method is shown in Figure 4.

Two forms of data that include the sensor data and the clock drawing image are generated for output once mCDT has been completed, as shown in Figure 4a. The clock drawing image, $I_{C}$, is intended to be of a clock face with numbers, hands and a contour. From the original 2400 × 1200 pixel drawing image at the CDT window, the clock drawing image $I_{C}$ is resized to 128 × 128 pixels. Time stamps t[n] in sec, x- and y- coordinates, x[n] and y[n] in pixels, and touch-events e[n] of the sensor data for the 128 × 128 drawing image have a sampling rate of 50 Hz with n being the index of a sample point. Each touch-event e[n] has a value such as −1, 0, 1; the assigned value of −1 designates the event ‘down’ for the screen being touched, 1 is for the event ‘up’ with the screen touch not continuing, and 0 is for the event ‘move’ with moving and touching on the screen continuing.

The sensor data x[n] and y[n], $c_{i}\leq n\leq c_{f}$ belonging to the contour in the clock drawing image $I_{C}$ are obtained using the touch-events e[n], $c_{i}\leq n\leq c_{f}$, where $c_{i}$ and $c_{f}$ are the start and the end indices of the contour, respectively, which can be estimated by the touch-event down-shifting from the event ‘down’ into the event ‘move’ and the touch-event up-shifting from the event ‘move’ into the event ‘up’, respectively. Besides, the touch-events e[n], $c_{i}<n<c_{f}$ between the touch-event down and up shiftings have to be successively stayed in the event ‘move’ for the longest time if such a period occurs more than once. In other words, the longest continuous sequence of 0 s in the touch-event e[n], starting with the digit −1 and ending with the digit 1, identifies itself as belonging to the contour in the clock drawing image $I_{C}$.

The contour image $I_{fc}$ is segmented from the clock drawing image $I_{C}$ using DeepC, the pre-trained models. Next, percentages $p_{fc}$ of the segmented image $I_{fc}$, matching to the corresponding portion of the clock drawing image $I_{C}$, is estimated by Equation (1), where $n(I_{C,c_{i}\leq n\leq c_{f}}\cap I_{fc})$ is the number of pixel coordinates that $I_{fc}$ and the contour image $I_{C,c_{i}\leq n\leq c_{f}}$ have in common; and $n(I_{C,c_{i}\leq n\leq c_{f}})$ is the total number of pixel coordinates in the sensor data belonging to the contour image.

(1) $$p_{fc}=\frac{n(I_{C,c_{i}\leq n\leq c_{f}}\cap I_{fc})}{n(I_{C,c_{i}\leq n\leq c_{f}})}$$

A modified clock drawing image $I_{C^{\prime }}$ is generated as shown in Figure 4b by redrawing the remaining part of the clock drawing image $I_{C}$ after excluding the sensor data x[n], y[n], t[n], $c_{i}\leq n\leq c_{f}$ belonging to the contour, and then by binarizing for the background to be black and for the number digits to be drawn white.

The hand image $I_{fh}$ is separately segmented from the clock drawing image $I_{C}$ using DeepH, the pre-trained models. Next, percentages $p_{fh}^{k}$, k=1,2 of the segmented images $I_{fh}$, matching to the corresponding portion of the clock drawing image $I_{C}$ are estimated by Equation (2), where $n(I_{C,h_{i}^{k}\leq n\leq h_{f}^{k}}\cap I_{fh})$ is the number of pixel coordinates that $I_{fh}$ and one of the hands images $I_{C,h_{i}^{k}\leq n\leq h_{f}^{k}}$, k=1,2 have in common; and $n(I_{C,h_{i}^{k}\leq n\leq h_{f}^{k}})$, k=1,2 is the total number of pixel coordinates in the sensor data belonging to one of the hands images.

(2) $$p_{fh}^{k}=\frac{n(I_{C,h_{i}^{k}\leq n\leq h_{f}^{k}}\cap I_{fh})}{n(I_{C,h_{i}^{k}\leq n\leq h_{f}^{k}})}$$

The sensor data x[n] and y[n], $h_{i}^{k}\leq n\leq h_{f}^{k}$, k=1,2 belonging to one of the hand and minute hands images in the modified clock drawing image $I_{C^{\prime }}$, are obtained using the touch events e[n], $h_{i}^{k}\leq n\leq h_{f}^{k}$, k=1,2, where $h_{i}^{k}$ and $h_{f}^{k}$ are the start and the end indices of the hand, respectively, which can be estimated by a touch-event down shifting from the event ‘down’ into the event ‘move’ and by the touch-event up shifting from the event ‘move’ into the event ‘up’, respectively. Here, the time stamps between $t[h_{i}^{k}]$ and $t[h_{f}^{k}]$ have to be overlapped with those between $t_{i}[j]$ and $t_{f}[j]$, j=1,2,…,N. If there is only one or no hands, so such a touch-event down or up shifting is not identified in the modified clock drawing image $I_{C^{\prime }}$, then the corresponding time stamps $t[h_{i}^{k}]$ and $t[h_{f}^{k}]$ are treated to have missing values, NAs.

The minimum and maximum x- and y- coordinates in pixels, $x_{\min}^{c}$ and $x_{\max}^{c}$, and $y_{\min}^{c}$ and $y_{\max}^{c}$, of the sensor data x[n] and y[n], $c_{i}\leq n\leq c_{f}$ are estimated as the boundary of the contour and formulated by Equations (3)–(6), respectively.

(3) $$x_{\min}^{c}=\underset{c_{i}\leq n\leq c_{f}}{\min}x[n]$$

(4) $$x_{\max}^{c}=\underset{c_{i}\leq n\leq c_{f}}{\max}x[n]$$

(5) $$y_{\min}^{c}=\underset{c_{i}\leq n\leq c_{f}}{\min}y[n]$$

(6) $$y_{\max}^{c}=\underset{c_{i}\leq n\leq c_{f}}{\max}y[n]$$

The x- and y- coordinates in pixels, $x_{mid}^{c}$ and $y_{mid}^{c}$ of the center point of the contour are defined by the bisecting point of the minimum and maximum x- and y- coordinates in pixels, $x_{\min}^{c}$ and $x_{\max}^{c}$, and $y_{\min}^{c}$ and $y_{\max}^{c}$, that are formulated by Equations (7) and (8), respectively:

(7) $$x_{mid}^{c}=\left(x_{\min}^{c}+x_{\max}^{c}\right)/2$$

(8) $$y_{mid}^{c}=\left(y_{\min}^{c}+y_{\max}^{c}\right)/2$$

Pre-estimated point positions $P_{k}=(x_{d}[k],y_{d}[k])$, k=1,2,…,12 of the clock number digits from 0 to 12 are evaluated as shown in Figure 4c by using the boundaries $x_{\min}^{c}$, $x_{\max}^{c}$, $y_{\min}^{c}$ and $y_{\max}^{c}$ of the contour along with the center point $x_{mid}^{c}$ and $y_{mid}^{c}$, where $x_{d}[k]$ and $y_{d}[k]$ are x- and y- coordinates in pixels of the kth digit number from 0 to 12. Table 3 summarizes the corresponding formula of each of the pre-estimated positions $P_{k}$, k=1,2,…,12.

Next, each of the number images $I_{C^{\prime }}^{j}$, j=1,2,…,N corresponding to a digit number is cropped out from the modified clock drawing image $I_{C^{\prime }}$ using the function findContours() of OpenCV2, where N is the total number of the digit images cropped out and j is the index sorted by the time stamps in ascending order. Here, the function findContours() can be used for finding the suburb contours of white objects from a black background [35].

The model DeepN classifies each of the number images $I_{C^{\prime }}^{j}$, j=1,2,…,N into one of 10 integer values ranging from 0 to 9, inclusive, and saves the identified integer in D[j]. At the same time, spatial data, $L_{ux}[j]$ $L_{uy}[j]$, $L_{dx}[j]$, and $L_{dy}[j]$, j=1,2,…,N, of the suburb contours and the corresponding time stamps $t_{i}[j]$ and $t_{f}[j]$, j=1,2,…,N are generated, where $L_{ux}[j]$ and $L_{uy}[j]$ are the upper left x- and y- coordinates in pixel of the jth suburb contour, respectively; $L_{dx}[j]$ and $L_{dy}[j]$ are the lower right x- and y- coordinates in pixels of the jth suburb contour, respectively; $t_{i}[j]\in t[n]$ and $t_{f}[j]\in t[n]$ are the corresponding initial and final time stamps of the sensor data belonging to the number image in the jth suburb contour, respectively, and the index j is sorted by the time stamp $t_{i}[j]$.

#### 2.4.1. Scoring on Criteria of Contour Parameter

Scoring on the circular shape, the closure (opening), and the size properness of the contour are evaluated by the percentage $p_{fc}$ of the segmented image $I_{fc}$ matching to the corresponding portion of the clock drawing image $I_{C}$, the maximum contour closure distance $d_{\max}^{c}$, and the ratio $A_{c}/W_{c}$ of the contour $A_{c}$ to the CDT window sizes $W_{c}$ as shown in Figure 4. The circular shape is identified if the value of the percentage $p_{fc}$ is larger than a given threshold $\theta_{c1}$. Scoring on the closure(opening) of the contour is evaluated by the first $p_{i}^{c}=(x[c_{i}],y[c_{i}])$ and the last $p_{f}^{c}=(x[c_{f}],y[c_{f}])$ sample points along with the contour sample points $p_{sh}^{k}=(x[c_{sh}^{k}],y[c_{sh}^{k}])$ at down or up shiftings in the touch events, that are shifting from ‘up’ to ‘move’ or from ‘move’ to ‘up’ at the time stamp $t[c_{sh}^{k}]$, $c_{i}<c_{sh}^{k}<c_{f}$. The closure of the contour is identified if it is greater than a given threshold $\theta_{c2}$, the maximum value $d_{\max}^{c}$ of $\left\|\left(p_{i}^{c},p_{f}^{c}\right)\right\|$, the distance between the first and the last contour sample points, and $\left\|\left(p_{sh}^{k},p_{sh}^{k+1}\right)\right\|$ the distances between the kth and the (k+1)th sample points shifting down or up in the touch events, where the index k is sorted by the time stamp of the sample points. The appropriateness of the contour size is evaluated by the ratio of the size of the contour to that of the CDT window. The contour size $A_{c}$ in pixels is calculated by the expression $A_{c}=(x_{\max}^{c}-x_{\min}^{c})(y_{\max}^{c}-y_{\min}^{c})$ using $x_{\min}^{c}$, $x_{\max}^{c}$, $y_{\min}^{c}$ and $y_{\max}^{c}$. The appropriate of the contour size is identified if the ratio $A_{c}/W_{c}$ is larger than a given threshold $\theta_{c3}$, where $W_{c}$ is the size in pixels of the CDT window.

#### 2.4.2. Scoring on Criteria of Numbers Parameter

**Figure 5:**
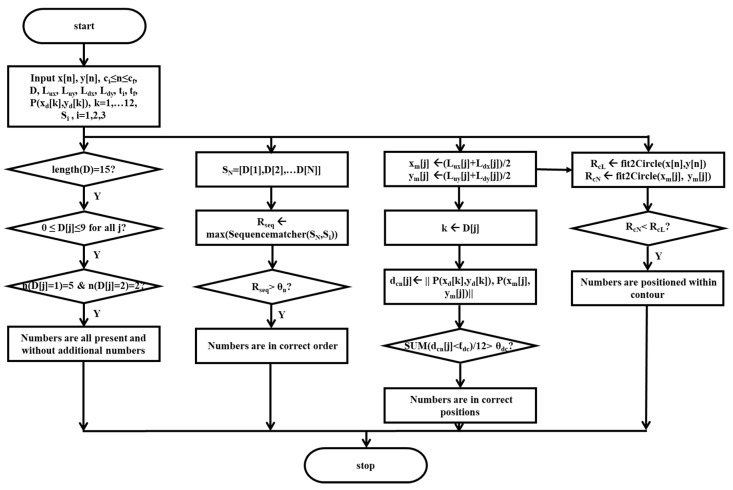
Flowcharts for scoring presence of all the numbers and no additional numbers; for scoring correctness of the order of the numbers; for scoring correctness of the positions of the numbers; and for scoring positioning of the numbers within the contour.

Figure 5 shows the flowcharts suggested in this study for scoring presence of all the numbers and no additional numbers, correctness of the order of the numbers, correctness of the positions of the numbers, and positioning of the numbers within the contour.

The presence of all the numbers and no additional numbers is evaluated by using the classified output D[j], j=1,2,…,N of DeepN for the cropped number images $I_{C^{\prime }}^{j}$, j=1,2,…,N and identified if the total number N is equal to 15, all the values in D[j], j=1,2,…,N are in the range from 0 to 9, and the count number of digits 1 and 2 are 5 and 2, respectively.

The correctness of the order of the numbers is evaluated by using the classified outputs D[j], j=1,2,…,N of DeepN for the cropped number images $I_{C^{\prime }}^{j}$, j=1,2,…,N. First of all, the number sequence $S_{N}=[\begin{array}{cccc} D[1] & D[2] & \ldots & D[N] \end{array}]$ is obtained and compared to reference number sequences considering general human habits. Here, three types of ordering in drawing numbers were considered as reference number sequences, such as drawing numbers starting from digits 1 through 12 in ascending order, starting from digit 12, and then digits 1 through 11 in ascending order, or starting from digits 12, 3, 6, and 9 and then inserting digits 1 through 11 in ascending order. Therefore, the reference number sequences considered were $S_{1}=[1,2,\ldots ,9,1,0,1,1,1,2]$, $S_{2}=[1,2,1,2,\ldots ,9,1,0,1,1]$, and $S_{3}=[1,2,3,6,9,1,2,4,5,7,8,1,0,1,1]$. The correctness of the order of the numbers is identified if the maximum value $R_{seq}=\underset{i=1,2,3}{\max}SequenceMatcher(S_{N},S_{i})$ of percentages of matched sequences between the number sequence $S_{N}$ and each of the reference number sequences $S_{1}$, $S_{2}$ and $S_{3}$ is greater than a given threshold $\theta_{n}$, where the function *SequenceMatcher*() is in the python library difflib [36].

The correctness of the position of the numbers is evaluated by the classified outputs D[j], $L_{ux}[j]$ $L_{uy}[j]$, $L_{dx}[j]$, and $L_{dy}[j]$, $t_{i}[j]$ and $t_{f}[j]$, j=1,2,…,N of DeepN for the cropped number images $I_{C^{\prime }}^{j}$, j=1,2,…,N as well as the predefined positions $P(x_{d}[k],y_{d}[k])$, k=1,2,…,12 of the number digits from 0 to 12. The center position $P(x_{m}[j],y_{m}[j])$, j=1,2,…,N of each of the cropped number images $I_{C}^{j}$, j=1,2,…,N is estimated by the bisecting point of the upper left point $P(L_{ux}[j],L_{uy}[j])$ and the lower right point $P(L_{dx}[j],L_{dy}[j])$, j=1,2,…,N, estimated by Equations (9) and (10).

(9) $$x_{m}[j]=\left(L_{ux}[j]+L_{dx}[j]\right)/2,\;j=1,2,\ldots ,N$$

(10) $$y_{m}[j]=\left(L_{uy}[j]+L_{dy}[j]\right)/2,\;j=1,2,\ldots ,N$$

The digit number k in D[j] is identified and the distance $d_{cn}[j]$ is estimated between the center position $P(x_{m}[j],y_{m}[j])$ of the jth cropped number images $I_{C^{\prime }}^{j}$ and the predefined position $P(x_{d}[k],y_{d}[k])$ corresponding to the identified digit number k in D[j]. Then, the correctness of the position of the numbers is identified if the percentage of the distances $d_{cn}[j]$, j=1,2,…,N; within a given limit, $\ell_{dc}$ is greater than a given value $\theta_{dc}$.

The positioning of the numbers within the contour is evaluated by using the center point $P(x_{m}[j],y_{m}[j])$, j=1,2,…,N of each of the cropped number images $I_{C^{\prime }}^{j}$, j=1,2,…,N and the contour sensor data, x[n] and y[n], $c_{i}\leq n\leq c_{f}$. A circle $F_{cL}$ is fitted to the contour sensor data, x[n] and y[n], $c_{i}\leq n\leq c_{f}$ using the least squares circle fitting algorithm [37], where the center point $P(x[n_{c}],y[n_{c}])$, $n_{c}\in \{n|c_{i}\leq n\leq c_{f}\}$ and the radius $R_{cL}$ of the fitted circle $F_{cL}$ are obtained. Similarly, a circle $F_{cN}$ is fitted to the center point $P(x_{m}[j],y_{m}[j])$, j=1,2,…,N using also the least squares circle fitting algorithm, where the center point $P(x[n_{cN}],y[n_{cN}])$ and the radius $R_{cN}$ of the fitted circle $F_{cN}$ are obtained. Then, the positioning of the numbers is identified if the radius $R_{cN}$ of the fitted circle $F_{cN}$ is smaller than the radius $R_{cL}$ of the fitted circle $F_{cL}$.

#### 2.4.3. Scoring on Criteria of Hands Parameter

**Figure 6:**
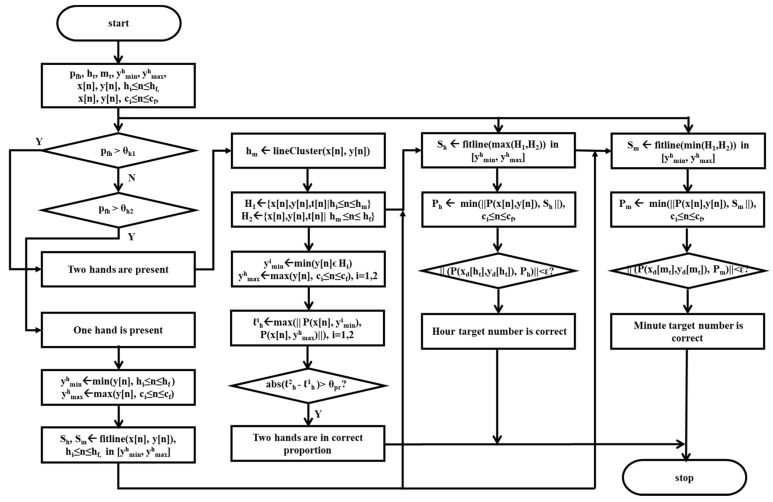
Flowcharts for scoring presence of one or two hands, correctness of the proportion of the hands, correctness of the hour target number, and correctness of the minute target number.

Figure 6 shows the flowchart suggested in this study for scoring the presence of two or one hand, correctness of the proportion of the hands, correctness of the hour target number, and correctness of the minute target number.

Presence of two or one hand is evaluated by the percentage $p_{fh}$ of the segmented image $I_{fh}$ matching to the corresponding portion of the clock drawing image $I_{C}$. The presence of two hands is identified if the value of the percentage $p_{fh}$ is larger than a given threshold $\theta_{h1}$. The presence of one hand is identified if the value of the percentage $p_{fh}$ is larger than a given threshold $\theta_{h2}$. Here, the value of the given threshold $\theta_{h2}$ is smaller than that of the given threshold $\theta_{h1}$ since DeepH is trained with images of the clock face with two hands so that the criteria of the two hands is included in the criteria of the one hand.

Indication of the correct proportion of hands is evaluated by using the hands sensor data x[n], y[n], t[n], and e[n]$h_{i}\leq n\leq h_{f}$ between the time stamps $t[h_{i}]$ and $t[h_{f}]$. Here, indication of the presence of two hands is a prerequisite of the indication of the correct proportion of hands. The hands sensor data x[n], y[n], t[n], and e[n]$h_{i}\leq n\leq h_{f}$ is divided into two sets $H_{1}=\{x[n],y[n],t[n],e[n]|h_{i}\leq n<h_{m}\}$ and $H_{2}=\{x[n],y[n],t[n],e[n]|h_{m}<n\leq h_{f}\}$ using a line clustering algorithm [38]. Here, the time stamp $t[h_{m}]$ $h_{i}<h_{m}<h_{f}$ is the time point of the intermission between the two sets $H_{1}$ and $H_{2}$. Then, the length $\ell_{h}^{1}$ of one hand is estimated as the maximum distance between $P(x[n],y_{\min}^{1})$, $h_{i}\leq n<h_{m}$ and $P(x[n],y_{\max}^{1})$, $h_{i}\leq n<h_{m}$ using the hand sensor data in the set $H_{1}$, where $y_{\min}^{1}$ and $y_{\max}^{1}$ are the minimum and maximum of y coordinates in the set $H_{1}$. Similarly, the length $\ell_{h}^{2}$ of another hand is estimated as the maximum distance between $P(x[n],y_{\min}^{2})$, $h_{m}<n\leq h_{f}$ and $P(x[n],y_{\max}^{2})$, $h_{m}<n\leq h_{f}$ using the hand sensor data in the set $H_{2}$, where $y_{\min}^{2}$ and $y_{\max}^{2}$ are the minimum and maximum of y coordinates in the set $H_{2}$. Finally, the indication of the correct proportion of hands is identified if the length difference $\Delta \ell_{h}=|\ell_{h}^{2}-\ell_{h}^{1}|$ between the lengths of one and another hand is larger than a given number $\theta_{pr}$.

Indication of the hour target number is evaluated by using the hands sensor data x[n], y[n], t[n], and e[n]$h_{i}\leq n\leq h_{f}$ between the time stamps $t[h_{i}]$ and $t[h_{f}]$. Two different cases are considered here; one is that the presence of two hands is identified, and the other is that the presence of only one hand is identified. For the first case, the hour hand sensor data $S_{h}$ with larger data size of the two sets $H_{1}$ and $H_{2}$ is fitted into a line and then the fitted line is extrapolated within a range $\left[y_{h,\min},y_{h,\max}\right]$ of y pixel coordinates, where $y_{h,\min}$ is the minimum value of y coordinates y[n], $h_{i}\leq n<h_{f}$ in the hands sensor data and $y_{h,\max}$, the maximum of y coordinates y[n], $c_{i}\leq n<c_{f}$ in the contour sensor data. For the second case, the whole hands sensor data is fitted into a line and then the fitted line is extrapolated within a range $\left[y_{h,\min},y_{h,\max}\right]$ of y pixel coordinates. Next, the closest point $P_{h}(x[n_{h}],y[n_{h}])$, $c_{i}\leq n<c_{f}$ of the contour sensor data to the extrapolated line is evaluated. Finally, the indication of the hour target number is identified if the point $P_{h}(x[n_{h}],y[n_{h}])$ is within a given range from a predefined pixel point $P(x_{d}[h_{t}],y_{d}[h_{t}])$ of the given hour target digit $h_{t}$.

Indication of minute target number is similarly evaluated by using the hands sensor data x[n], y[n], t[n], and e[n]$h_{i}\leq n\leq h_{f}$ between the time stamps $t[h_{i}]$ and $t[h_{f}]$. Two different cases are considered here; one is that the presence of two hands is identified, and the other is that the presence of only one hand is identified. For the first case, the minute hand sensor data $S_{m}$ with smaller data size of the two sets $S_{1}$ and $S_{2}$ is fitted into a line and then the fitted line is extrapolated within a range $\left[y_{h,\min},y_{h,\max}\right]$ of y pixel coordinates, where $y_{h,\min}$ is the minimum value of y coordinates y[n], $h_{i}\leq n<h_{f}$ in the hands sensor data, and $y_{h,\max}$ is the maximum of y coordinates y[n], $c_{i}\leq n<c_{f}$ in the contour sensor data. For the second case, the whole hands sensor data is fitted into a line and then the fitted line is extrapolated within a range $\left[y_{h,\min},y_{h,\max}\right]$ of y pixel coordinates. Next, the closest point $P(x[n_{m}],y[n_{m}])$, $c_{i}\leq n<c_{f}$ of the contour sensor data to the extrapolated line is evaluated. Finally, the indication of a minute target number is identified if the point $P(x[n_{m}],y[n_{m}])$ is within a given range from a predefined pixel point $P(x_{d}[m_{t}],y_{d}[m_{t}])$ of the given minute target digit $m_{t}$. Here, the point $P(x[n_{m}],y[n_{m}])$ has to be the same as the point $P(x[n_{h}],y[n_{h}])$ if the indication of the hour target number is already identified.

#### 2.4.4. Scoring Criteria of Center Parameter

Presence or inference of the center point of the clock face in the drawing image $I_{C}$ is identified, as shown in Figure 4 if the presence of two or one hand is identified. Also, presence or inference of the center is identified if there is a data point within a given range from the center point $P(x_{mid}^{c},y_{mid}^{c})$.

#### 2.4.5. Assignment of Scores

Table 4 lists the conditions for assigning scores for each parameter in mCDT, where the heuristic values of all the thresholds used in this study were summarized at the footnote.

The score of the contour parameter is via the percentage $p_{fc}$, the maximum contour closure distance $d_{\max}^{c}$, and the ratio of the contour to the CDT window sizes $A_{c}/W_{c}$. The score of the circular contour is a 1 if the percentage $p_{fc}$ is greater than a given threshold $\theta_{c1}$; the score of the closure contour is a 1 if the maximum contour closure distance $d_{\max}^{c}$ is greater than a given threshold $\theta_{c2}$; the score of the appropriate sized contour is a 1 if the ratio of the contour to the CDT window sizes $A_{c}/W_{c}$ is greater than a given threshold $\theta_{c3}$.

The score of the numbers parameter is determined by the contour sensor data, x[n] and y[n], $c_{i}\leq n\leq c_{f}$, the outputs, D[j], $L_{ux}[j]$ $L_{uy}[j]$, $L_{dx}[j]$, $L_{dy}[j]$, $t_{i}[j]$ and $t_{f}[j]$, j=1,2,…,N of DeepN, the reference number sequences $S_{i}$, i=1,2,3, and the predefined number positions $P(x_{d}[k],y_{d}[k])$, k=1,2,…,12 of the number digits from 0 to 12. There are four criteria on the numbers parameter, such as the presence of all the numbers and no additional numbers, the correctness of the order of the numbers, the correctness of the position of the numbers, and the positioning of the numbers within the contour. The score of the presence of all the numbers and no additional numbers is a 1 if the total number N is equal to 15, all the values in D[j], j=1,2,…,N are in the range from 0 to 9, and the count number of digits 1 and 2 are 5 and 2, respectively. The score of the correctness of the order of the numbers is a 1 if the maximum value $R_{seq}$ of percentages of matched sequence between the number sequence $S_{N}$ and each of the reference number sequences $S_{1}$, $S_{2}$ and $S_{3}$ is greater than a given threshold $\theta_{n}$. The score of the correctness of the position of the numbers is a 1 if the percentage of the distances $d_{cn}[j]$, j=1,2,…,N within a given limit $\ell_{dc}$ is greater than a given value $\theta_{dc}$, where the distance $d_{cn}[j]$ is estimated between the jth center position $P((L_{ux}[j]+L_{dx}[j])/2,(L_{uy}[j]+L_{dy}[j])/2)$ and the predefined position $P(x_{d}[k],y_{d}[k])$ corresponding to the identified digit number k in D[j]. The score of the positioning of the numbers within the contour is a 1 if the radius $R_{cN}$ is smaller than the radius $R_{cL}$, where the radius $R_{cN}$ is of the fitted circle $F_{cN}$ to the center point $P(x_{m}[j],y_{m}[j])$, j=1,2,…,15 and the radius $R_{cL}$ is of the fitted circle $F_{cL}$ to the contour sensor data, x[n] and y[n], $c_{i}\leq n\leq c_{f}$. The score of criteria on the numbers parameter is the sum of the scores of the presence of all the numbers and no additional numbers, the correctness of the order of the numbers, the correctness of the position of the numbers, and the positioning of the numbers within the contour.

The score of the hands parameter is determined by the percentage $p_{fh}$, the linearly clustered sets, $H_{1}$ and $H_{2}$ of the hands sensor data x[n] and y[n], $h_{i}\leq n\leq h_{f}$, the contour sensor data x[n] and y[n], $c_{i}\leq n\leq c_{f}$, the point $P(x[n_{h}],y[n_{h}])$ in the range $\left[y_{\min}^{i},y_{\max}^{h}\right]$, i=1,2 where $y_{\min}^{i}$ is the minimum of y coordinates y[n] in $H_{i}$, i=1,2 and $y_{h,\max}$ is the maximum of y coordinates y[n], $c_{i}\leq n<c_{f}$. There are four criteria on hands parameter, such as the presence of two or one hand, the indication of the correct proportion of hands, the indication of the hour target number, the indication of the minute target number, and the positioning of the numbers within the contour. The score of the presence of two hands or one hand is a 2 if the value of the percentage $p_{fh}$ is larger than a given threshold, $\theta_{h1}$; a 1 if the value of the percentage $p_{fh}$ is larger than a given threshold $\theta_{h2}$. The score of the indication of the correct proportion of hands is a 1 if the value of the percentage $p_{fh}$ is larger than a given threshold $\theta_{h1}$ and the size difference $\Delta \ell_{h}$ of the fitted and extrapolated lines in data between $H_{1}$ and $H_{2}$ is larger than a given number $\theta_{pr}$, where $H_{1}$ and $H_{2}$ are the two sets divided by a line cluster algorithm on the hands sensor data, x[n] and y[n], $h_{i}\leq n\leq h_{f}$. The score of the indication of the hour target number is a 1 if the point $P(x[n_{h}],y[n_{h}])$ is within a given range of a predefined pixel point of the given hour target, where the point $P(x[n_{h}],y[n_{h}])$ is obtained by estimating the closest one of the extrapolated hands sensor data spatially to the contour sensor data within the range $\left[y_{h,\min},y_{h,\max}\right]$. The score of the indication of the minute target number is a 1 if the point $P(x[n_{m}],y[n_{m}])$ is within a given range of predefined pixel points of the given minute target, where the point $P(x[n_{m}],y[n_{m}])$ is obtained by estimating the closest of the extrapolated hands sensor data spatially to the contour sensor data within the range $\left[y_{h,\min},y_{h,\max}\right]$. Here, the point $P(x[n_{m}],y[n_{m}])$ has to be the same as the point $P(x[n_{h}],y[n_{h}])$ if the indication of the hour target number is already identified. The score of the criteria on the hands parameter is the sum of the scores of the presence of two hands or one hand, the indication of the correct proportion of hands, the indication of the hour target number, the indication of the minute target number, and the positioning of the numbers within the contour.

The score of the criteria of the center parameter is determined by the center point $P(x_{mid}^{c},y_{mid}^{c})$ of the contour sensor data, x[n] and y[n], $c_{i}\leq n\leq c_{f}$. The score of the presence or the inference of the center of the clock face is a 1 if the presence of two or one hands is identified, or if there is a data point within a given range from the center point $P(x_{mid}^{c},y_{mid}^{c})$.

## 3. Results

### 3.1. Scoring on Criteria of Contour Parameter

**Figure 7:**
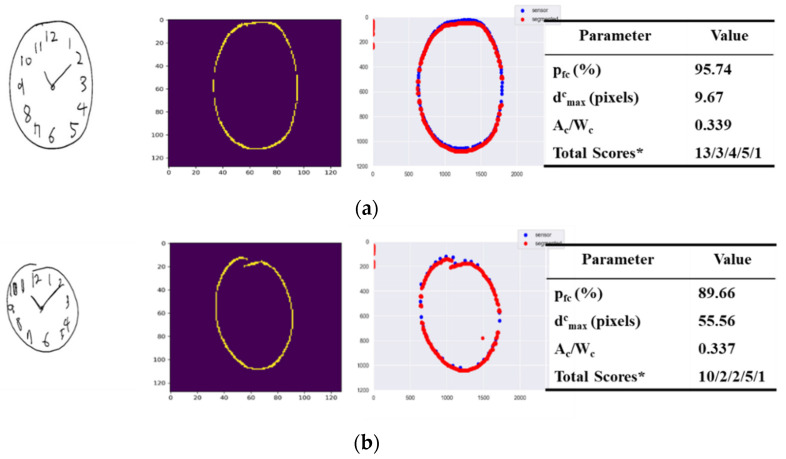

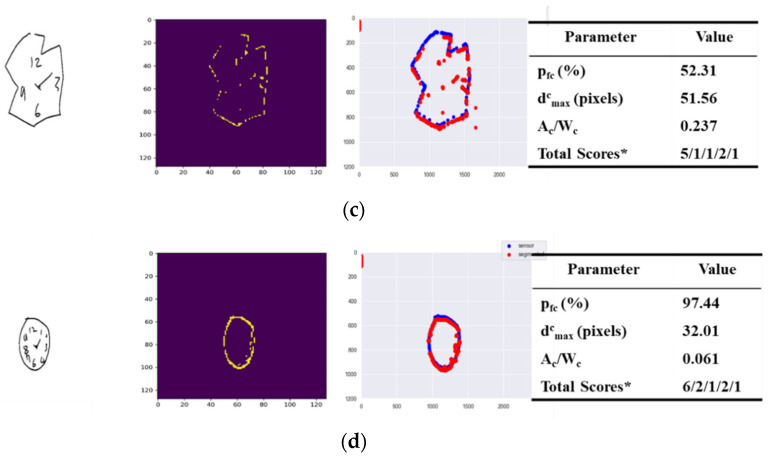
Four cases of original drawings (**left**) along with their segmented images (**middle**) produced by the pre-trained model, DeepC, and their corresponding velocity graphs (**right**). Four representative examples demonstrating how contours in original images are detected and classified in their types and sizes, showing the original image (first column) with the segmented image (second column), the overlapping image (third column) and the corresponding parameter values (fourth column) of each examples; (**a**) the case in which the original image is of a closed circular contour sized appropriately; (**b**) the case in which the original image is of a circular contour sized appropriately, but not wholly closed; (**c**) the case in which the original image is of an appropriately sized but neither closed nor circular contour; and (**d**) the case in which the original image is of a closed circular contour but sized not appropriately. * Total score/score of contour/score of numbers/score of hands/score of center.

Figure 7 depicts separate examples of original drawings (first column) each with the segmented image (second column) perceived by DeepC for a detected contour, the overlapping image (third column) of the original and the segmented images and the corresponding parameter values including the total score estimated.

In Figure 7a where the original image is of a closed circular contour sized appropriately, the segmented image has the estimated percentage $p_{fc}$ of 95.74%, the maximum contour closure distance has the evaluated pixel value $d_{\max}^{c}$ of 9.67 pixels, and the ratio of the contour to the CDT window size has the estimated value $A_{c}/W_{c}$ of 0.339. Both the closure and circular of the contour were evaluated to be one, as $p_{fc}$ was greater than the 75.00 score for $\theta_{c1}$, a threshold heuristically set, and $d_{\max}^{c}$ was less than the 50.00 score for $\theta_{c2}$, a threshold heuristically set. The size of the contour was also evaluated to be one, as $A_{c}/W_{c}$ was greater than the 0.1 score for $\theta_{c3}$, a threshold heuristically set. Therefore, the total score of the contour parameter was evaluated to be three.

Figure 7b has an original drawing image of a circular contour sized appropriately, but not wholly closed. The segmented image has the estimated percentage $p_{fc}$ of 89.66%, the maximum contour closure distance has the evaluated pixel value $d_{\max}^{c}$ of 55.56 pixels, and the ratio of the contour to the CDT window sizes has the estimated value $A_{c}/W_{c}$ of 0.337. The closure of the contour was evaluated to be zero, as $d_{\max}^{c}$ was greater than the 50.00 score for $\theta_{c2}$; however, both the circular and the size were gauged to be one, as the estimated percentage $p_{fc}$ was greater than the 75.00 score for $\theta_{c1}$ and $A_{c}/W_{c}$ was greater than the 0.1 score for $\theta_{c3}$. Therefore, the total score of the contour parameter was evaluated to be two.

Figure 7c shows an example of an original drawing of an appropriately sized, but neither closed nor circular, contour. The segmented image has the estimated percentage $p_{fc}$ of 52.31%, the maximum contour closure distance has the evaluated pixel value $d_{\max}^{c}$ of 51.56 pixels, and the ratio of the contour to the CDT window sizes has the estimated value $A_{c}/W_{c}$ of 0.237. Both the closure and circular of the contour were evaluated to be zero, as $p_{fc}$ was not greater than the 75.00 score for $\theta_{c1}$ and $d_{\max}^{c}$ was greater than the 50.00 score for $\theta_{c2}$; however, the size was gauged to be one, as $A_{c}/W_{c}$ was greater than the 0.1 score for $\theta_{c3}$. Therefore, the total score of the contour parameter was evaluated to be one.

Finally, the original drawing image of Figure 7d depicts an example of a closed circular contour, but not sized appropriately. The segmented image has the estimated percentage $p_{fc}$ of 97.44%, the maximum contour closure distance has the evaluated pixel value $d_{\max}^{c}$ of 32.01 pixels, and the ratio of the contour to the CDT window sizes has the estimated value $A_{c}/W_{c}$ of 0.061. Both the closure and circular of the contour were evaluated to be one, as $p_{fc}$ was greater than the 75.00 score for $\theta_{c1}$ and $d_{\max}^{c}$ was less than the 50.00 score for $\theta_{c2}$; however, the size was gauged to be zero, as $A_{c}/W_{c}$ was less than the 0.1 score for $\theta_{c3}$. The total score of the contour parameter was evaluated to be.

### 3.2. Scoring on Criteria of Number Parameter

**Figure 8:**
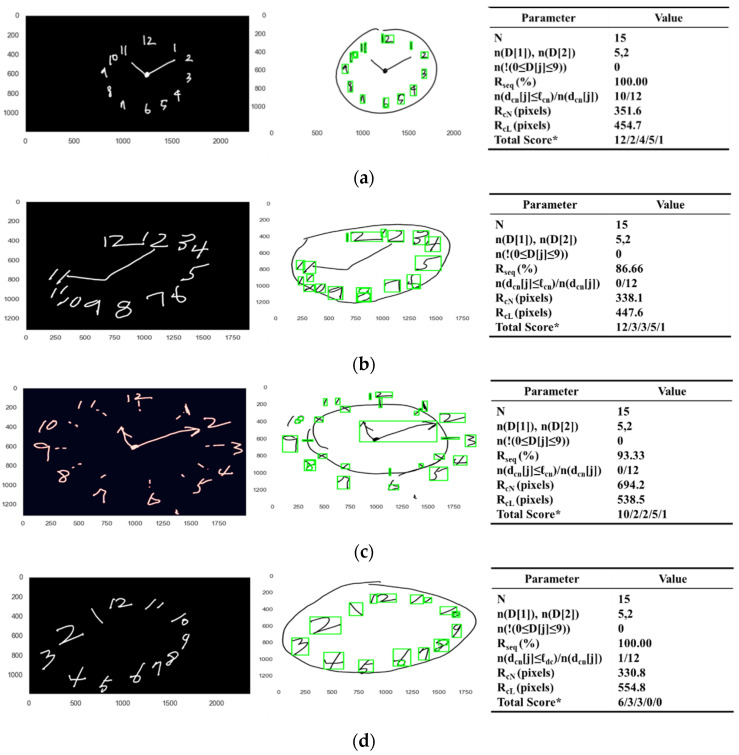

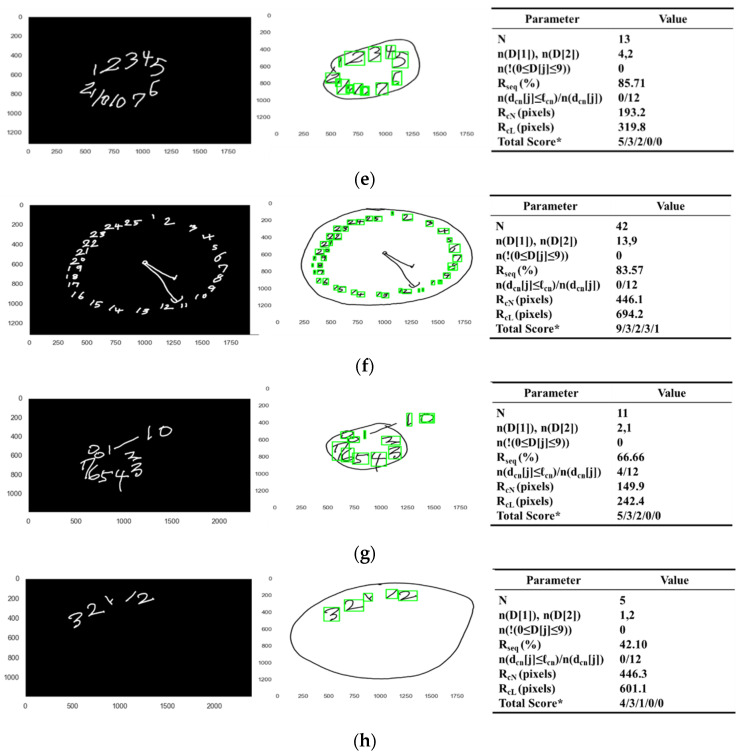
Eight representative examples demonstrating how numbers in original images are detected and classified in their orders and positions, showing the binarized image (**left**), the original image with the cropped areas for the numbers (**middle**), and the corresponding parameter values (**right**) of each example; (**a**) the case in which all the numbers without any additional numbers are present in the correct orders and the proper positions within the contour; (**b**) the case in which all the numbers without any additional numbers are present in the correct orders within the contour but not in proper positions; (**c**) the case in which all the numbers without any additional numbers are present in the correct orders but neither in the proper positions nor within the contour; (**d**) the case in which all the numbers without any additional numbers are present in the correct orders within the contour but not in the proper positions; (**e**) the case in which some numbers are missing and the presented numbers are not in proper positions but mostly in correct order within the contour; (**f**) the case in which there are additional numbers not belonging to a clock but the numbers are in correct orders within the contour; (**g**) the case in which some numbers are missing and the presented numbers are not in proper positions but mostly in correct order within the contour; and (**h**) the case in which many numbers are missing and the presented numbers are not in proper positions and correct order but within the contour. * Total score/score of contour/score of numbers/score of hands/score of center.

In eight representative examples, Figure 8 demonstrates how numbers in original images are detected and classified in their orders and positions, showing the binarized image (left), the original image with the cropped areas for the numbers (middle), and the corresponding parameter values (right) of each example.

Figure 8a displays the case in which all the numbers without any additional numbers are present in the correct orders and the proper positions within the contour. The classified output D[j], j=1,2,…,N of DeepN, the pretrained model, was identified to have no missing values, since the total umber N was equal to 15, and the classified output D[j], j=1,2,…,N was in the range from zero to nine, that is 0≤D[j]≤9 for all j, and the numbers n(D[j]=1) and n(D[j]=2) were five and two, respectively. Therefore, the presence of all the numbers and no additional numbers was evaluated to be one. The maximum ratio $R_{seq}$ in percentage between the number sequence $S_{N}$ and the reference sequences $S_{i}$ i=1,2,3, was evaluated to be 100.00%. Therefore, the correctness of the order of the numbers was evaluated to be one, since the value 100.00 of the maximum ratio $R_{seq}$ was greater than 65.00, the heuristically given value of $\theta_{n}$. The ratio *n*($d_{cn}[j]$ ≤ $\ell_{dc}$)/*n*($d_{cn}[j]$) of the distances $d_{cn}[j]$, j=1,2,…,12 within 100.00 pixels, a given limit $\ell_{dc}$, was greater than 0.65, a heuristically given value $\theta_{dc}$, where the evaluated distance $d_{cn}[j]$ for each of the digit numbers k in D[j] in pixels was specifically 46.0, 66.9, 27.5, 32.0, 33.0, 16.6, 119.0, 123.0, 38.0, 51.1, 40.7 and 22.6. Therefore, the correctness of the position of the numbers was evaluated to be one. The radius $R_{cL}$ of the fitted circle $F_{cL}$ to the contour sensor data was obtained to be 454.7 pixels. The radius $R_{cN}$ of the fitted circle $F_{cN}$ to the center point $P(x_{m}[j],y_{m}[j])$, j=1,2,…,12 of each of the cropped number images $I_{C}^{j}$, j=1,2,…,N was obtained to be 351.6 pixels. Now, the positioning of the numbers within the contour was evaluated to be one, since the radius $R_{cN}$ of the fitted circle $F_{cN}$ is smaller than the radius $R_{cL}$ of the fitted circle $F_{cL}$. Finally, the total score of the numbers parameter was evaluated to be four.

Figure 8b displays the case in which all the numbers without any additional numbers are present in the correct orders within the contour but not in proper positions. The classified output D[j], j=1,2,…,N of DeepN, the pretrained model, was identified to have no missing values, since the total number N was equal to 15, and the classified output D[j], j=1,2,…,N was in the range from zero to nine, that is 0≤D[j]≤9 for all j, and the numbers n(D[j]=1) and n(D[j]=2) were five and two, respectively. Therefore, the presence of all the numbers and no additional numbers was evaluated to be one. The maximum ratio $R_{seq}$ in percentage between the number sequence $S_{N}$ and the reference sequences $S_{i}$ i=1,2,3, was evaluated to be 86.66%. Therefore, the correctness of the order of the numbers was evaluated to be one, since the value 86.66 of the maximum ratio $R_{seq}$ was greater than 65.00, the heuristically given value of $\theta_{n}$. The ratio *n*($d_{cn}[j]$ ≤ $\ell_{dc}$)/*n*($d_{cn}[j]$) of the distances $d_{cn}[j]$, j=1,2,…,12 within 100.00 pixels, a given limit $\ell_{dc}$, was less than 0.65, a heuristically given value $\theta_{dc}$, where the evaluated distance $d_{cn}[j]$ in pixels for each of the digit numbers k in D[j] was specifically 243.0, 367.8, 366.93, 594.4, 518.2, 398.0, 491.1, 365.9, 418.2, 404.0, 628.2 and 143.6. Therefore, the correctness of the position of the numbers was evaluated to be zero. The radius $R_{cL}$ of the fitted circle $F_{cL}$ to the contour sensor data was obtained to be 447.6 pixels. The radius $R_{cN}$ of the fitted circle $F_{cN}$ to the center point $P(x_{m}[j],y_{m}[j])$, j=1,2,…,12 of each of the cropped number images $I_{C}^{j}$, j=1,2,…,N was obtained to be 338.1 pixels. Now, the positioning of the numbers within the contour was evaluated to be one, since the radius $R_{cN}$ of the fitted circle $F_{cN}$ is smaller than the radius $R_{cL}$ of the fitted circle $F_{cL}$. Finally, the total score of the numbers parameter was evaluated to be three.

Figure 8c displays the case in which all the numbers without any additional numbers are present in the correct orders, but not in the proper positions nor within the contour. The classified output D[j], j=1,2,…,N of DeepN, the pretrained model, was identified to have no missing values, since the total number N was equal to 15, and the classified output D[j], j=1,2,…,N was in the range from zero to nine, that is 0≤D[j]≤9 for all j, and the numbers n(D[j]=1) and n(D[j]=2) were five and two, respectively. Therefore, the presence of all the numbers and no additional numbers was evaluated to be one. The maximum ratio $R_{seq}$ in percentage between the number sequence $S_{N}$ and the reference sequences $S_{i}$ i=1,2,3, was evaluated to be 93.33%. Therefore, the correctness of the order of the numbers was evaluated to be one, since the value 93.33 of the maximum ratio $R_{seq}$ was greater than 65.00, the heuristically given value of $\theta_{n}$. The ratio *n*($d_{cn}[j]$ ≤ $\ell_{dc}$)/*n*($d_{cn}[j]$) of the distances $d_{cn}[j]$, j=1,2,…,12 within 100.00 pixels, a given limit $\ell_{dc}$, was less than 0.65, a heuristically given value $\theta_{dc}$, where the evaluated distance $d_{cn}[j]$ for each of the digit numbers k in D[j] was specifically 147.7, 170.7, 214.2, 364.0, 369.4, 139.1, 114.3, 226.4, 157.0, 242.5, 224.0 and 127.6. Therefore, the correctness of the position of the numbers was evaluated to be zero. The radius $R_{cL}$ of the fitted circle $F_{cL}$ to the contour sensor data was obtained to be 538.5 pixels. The radius $R_{cN}$ of the fitted circle $F_{cN}$ to the center point $P(x_{m}[j],y_{m}[j])$, j=1,2,…,12 of each of the cropped number images $I_{C}^{j}$, j=1,2,…,N was obtained to be 694.2 pixels. Now, the positioning of the numbers within the contour was evaluated to be zero, since the radius $R_{cN}$ of the fitted circle $F_{cN}$ is larger than the radius $R_{cL}$ of the fitted circle $F_{cL}$. Finally, the total score of the numbers parameter was evaluated to be two.

Figure 8d displays the case in which all the numbers without any additional numbers are present in the correct orders within the contour, but not in the proper positions. The classified output D[j], j=1,2,…,N of DeepN, the pretrained model, was identified to have no missing values, since the total number N was equal to 15, and the classified output D[j], j=1,2,…,N was in the range from zero to nine, that is 0≤D[j]≤9 for all j, and the numbers n(D[j]=1) and n(D[j]=2) were five and two, respectively. Therefore, the presence of all the numbers and no additional numbers was evaluated to be one. The maximum ratio $R_{seq}$ in percentage between the number sequence $S_{N}$ and the reference sequences $S_{i}$ i=1,2,3, was evaluated to be 100.00%. Therefore, the correctness of the order of the numbers was evaluated to be one, since the value 100.00 of the maximum ratio $R_{seq}$ was greater than 65.00, the heuristically given value of $\theta_{n}$. The ratio *n*($d_{cn}[j]$ ≤ $\ell_{dc}$)/*n*($d_{cn}[j]$) of the distances $d_{cn}[j]$, j=1,2,…,12 within 100.00 pixels, a given limit $\ell_{dc}$, was less than 0.65, a heuristically given value $\theta_{dc}$, where the evaluated distance $d_{cn}[j]$ for each of the digit numbers k in D[j] was specifically 618.19, 1106.19, 1408.42, 931.44, 378.00, 185.95, 630.61, 1079.10, 1381.07, 1314.39, 720.95 and 58.69. Therefore, the correctness of the position of the numbers was evaluated to be zero. The radius $R_{cL}$ of the fitted circle $F_{cL}$ to the contour sensor data was obtained to be 554.8 pixels. The radius $R_{cN}$ of the fitted circle $F_{cN}$ to the center point $P(x_{m}[j],y_{m}[j])$, j=1,2,…,12 of each of the cropped number images $I_{C}^{j}$, j=1,2,…,N was obtained to be 330.8 pixels. Now, the positioning of the numbers within the contour was evaluated to be one, since the radius $R_{cN}$ of the fitted circle $F_{cN}$ is smaller than the radius $R_{cL}$ of the fitted circle $F_{cL}$. Finally, the total score of the numbers parameter was evaluated to be three.

Figure 8e displays the case in which some numbers are missing and the presented numbers are not in proper positions, but mostly in correct order within the contour. The classified output D[j], j=1,2,…,N of DeepN, the pretrained model, was identified to have some missing values, since the total number N was equal to 13, and the classified output D[j], j=1,2,…,N was in the range from zero to nine, that is 0≤D[j]≤9 for all j, and the numbers n(D[j]=1) and n(D[j]=2) were four and two, respectively. Therefore, the presence of all the numbers and no additional numbers was evaluated to be zero. The maximum ratio $R_{seq}$ in percentage between the number sequence $S_{N}$ and the reference sequences $S_{i}$ i=1,2,3, was evaluated to be 85.71%. Therefore, the correctness of the order of the numbers was evaluated to be one, since the value 85.71 of the maximum ratio $R_{seq}$ was greater than 65.00, the heuristically given value of $\theta_{n}$. The ratio *n*($d_{cn}[j]$ ≤ $\ell_{dc}$)/*n*($d_{cn}[j]$) of the distances $d_{cn}[j]$, j=1,2,…,12 within 100.00 pixels, a given limit $\ell_{dc}$, was less than 0.65, a heuristically given value $\theta_{dc}$, where the evaluated distance $d_{cn}[j]$ for each of the digit numbers k in D[j] was specifically 497.8, 462.5, 350.7, 399.3, 415.1, 254.5, 208.5, 1037.8, 836.2, 792.1, 743.6 and 952.1. Therefore, the correctness of the position of the numbers was evaluated to be zero. The radius $R_{cL}$ of the fitted circle $F_{cL}$ to the contour sensor data was obtained to be 319.8 pixels. The radius $R_{cN}$ of the fitted circle $F_{cN}$ to the center point $P(x_{m}[j],y_{m}[j])$, j=1,2,…,12 of each of the cropped number images $I_{C}^{j}$, j=1,2,…,N was obtained to be 193.2 pixels. Now, the positioning of the numbers within the contour was evaluated to be one, since the radius $R_{cN}$ of the fitted circle $F_{cN}$ is smaller than the radius $R_{cL}$ of the fitted circle $F_{cL}$. Finally, the total score of the numbers parameter was evaluated to be two.

Figure 8f displays the case in which there are additional numbers not belonging to a clock but the numbers are in correct orders within the contour. The classified output D[j], j=1,2,…,N of DeepN the pretrained model was identified to have some additional numbers, since the total number N was equal to 42, and the classified output D[j], j=1,2,…,N was in the range from zero to nine, that is 0≤D[j]≤9 for all j, and the numbers n(D[j]=1) and n(D[j]=2) was thirteen and nine, respectively. Therefore, the presence of all the numbers and no additional numbers was evaluated to be zero. The maximum ratio $R_{seq}$ in percentage between the number sequence $S_{N}$ and the reference sequences $S_{i}$ i=1,2,3, were evaluated to be 83.57%. Therefore, the correctness of the order of the numbers was evaluated to be one, since the value 83.57 of the maximum ratio $R_{seq}$ was greater than 65.00, the heuristically given value of $\theta_{n}$. The ratio *n*($d_{cn}[j]$ ≤ $\ell_{dc}$)/*n*($d_{cn}[j]$) of the distances $d_{cn}[j]$, j=1,2,…,12 within 100.00 pixels, a given limit $\ell_{dc}$, was less than 0.65, a heuristically given value $\theta_{dc}$, where the evaluated distance $d_{cn}[j]$ for each of the digit numbers k in D[j] was specifically 376.5, 483.4, 432.1, 636.2, 743.4, 856.4, 947.7, 1056.9, 1171.4, 1113.0, 826.2 and 837.7. Therefore, the correctness of the position of the numbers was evaluated to be zero. The radius $R_{cL}$ of the fitted circle $F_{cL}$ to the contour sensor data was obtained to be 694.2 pixels. The radius $R_{cN}$ of the fitted circle $F_{cN}$ to the center point $P(x_{m}[j],y_{m}[j])$, j=1,2,…,12 of each of the cropped number images $I_{C}^{j}$, j=1,2,…,N was obtained to be 446.1 pixels. Now, the positioning of the numbers within the contour was evaluated to be one, since the radius $R_{cN}$ of the fitted circle $F_{cN}$ was smaller than the radius $R_{cL}$ of the fitted circle $F_{cL}$. Finally, the total score of the numbers parameter was evaluated to be two.

Figure 8g displays the case in which some numbers are missing and the presented numbers are not in proper positions, but mostly in correct order within the contour. The classified output D[j], j=1,2,…,N of DeepN, the pretrained model, was identified to have some additional numbers, since the total number N was equal to 11, and the classified output D[j], j=1,2,…,N was in the range from zero to nine, that is 0≤D[j]≤9 for all j, and the numbers n(D[j]=1) and n(D[j]=2) were two and one, respectively. Therefore, the presence of all the numbers and no additional numbers was evaluated to be zero. The maximum ratio $R_{seq}$ in percentage between the number sequence $S_{N}$ and the reference sequences $S_{i}$ i=1,2,3, were evaluated to be 66.66%. Therefore, the correctness of the order of the numbers was evaluated to be one, since the value 66.66 of the maximum ratio $R_{seq}$ was greater than 65.00, the heuristically given value of $\theta_{n}$. The ratio *n*($d_{cn}[j]$ ≤ $\ell_{dc}$)/*n*($d_{cn}[j]$) of the distances $d_{cn}[j]$, j=1,2,…,12 within 100.00 pixels, a given limit $\ell_{dc}$, was less than 0.65, a heuristically given value $\theta_{dc}$, where the evaluated distance $d_{cn}[j]$ for each of the digit numbers k in D[j] was specifically 87.7, 68.9, 56.1, 78.0, 163.0, 190.1, 232.8, 265.3, 894.3, 860.6, 802.5 and 990.8. Therefore, the correctness of the position of the numbers was evaluated to be zero. The radius $R_{cL}$ of the fitted circle $F_{cL}$ to the contour sensor data was obtained to be 242.4 pixels. The radius $R_{cN}$ of the fitted circle $F_{cN}$ to the center point $P(x_{m}[j],y_{m}[j])$, j=1,2,…,12 of each of the cropped number images $I_{C}^{j}$, j=1,2,…,N was obtained to be 149.9 pixels. Now, the positioning of the numbers within the contour was evaluated to be one, since the radius $R_{cN}$ of the fitted circle $F_{cN}$ is smaller than the radius $R_{cL}$ of the fitted circle $F_{cL}$. Finally, the total score of the numbers parameter was evaluated to be two.

Figure 8h displays the case in which many numbers are missing and the presented numbers are not in proper positions and correct order, but within the contour. The classified output D[j], j=1,2,…,N of DeepN the pretrained model was identified to have some missing numbers, since the total number N is equal to five, and the classified output D[j], j=1,2,…,N was in the range from zero to nine, that is 0≤D[j]≤9 for all j, and the numbers n(D[j]=1) and n(D[j]=2) were one and two, respectively. Therefore, the presence of all the numbers and no additional numbers was evaluated to be zero. The maximum ratio $R_{seq}$ in percentage between the number sequence $S_{N}$ and the reference sequences, $S_{i}$ i=1,2,3, were evaluated to be 42.10%. Therefore, the correctness of the order of the numbers was evaluated to be zero since the value 42.10 of the maximum ratio $R_{seq}$ was less than 65.00, the heuristically given value of $\theta_{n}$. The ratio *n*($d_{cn}[j]$ ≤ $\ell_{dc}$)/*n*($d_{cn}[j]$) of the distances $d_{cn}[j]$, j=1,2,…,12 within 100.00 pixels, a given limit $\ell_{dc}$, was less than 0.65, a heuristically given value $\theta_{dc}$, where the evaluated distance $d_{cn}[j]$ for each of the digit numbers k in D[j] was specifically 545.5, 920.4, 1167.1, 1739.7, 1644.2, 1464.5, 1338.6, 1095.1, 715.6, 630.7, 734.1 and 153.0. Therefore, the correctness of the position of the numbers was evaluated to be zero. The radius $R_{cL}$ of the fitted circle $F_{cL}$ to the contour sensor data was obtained to be 601.1 pixels. The radius $R_{cN}$ of the fitted circle $F_{cN}$ to the center point $P(x_{m}[j],y_{m}[j])$, j=1,2,…,15 of each of the cropped number images $I_{C}^{j}$, j=1,2,…,15 was obtained to be 446.3 pixels. Now, the positioning of the numbers within the contour was evaluated to be one, since the radius $R_{cN}$ of the fitted circle $F_{cN}$ is smaller than the radius $R_{cL}$ of the fitted circle $F_{cL}$. Finally, the total score of the numbers parameter was evaluated to be one.

### 3.3. Scoring on Criteria of Hand Parameter

**Figure 9:**
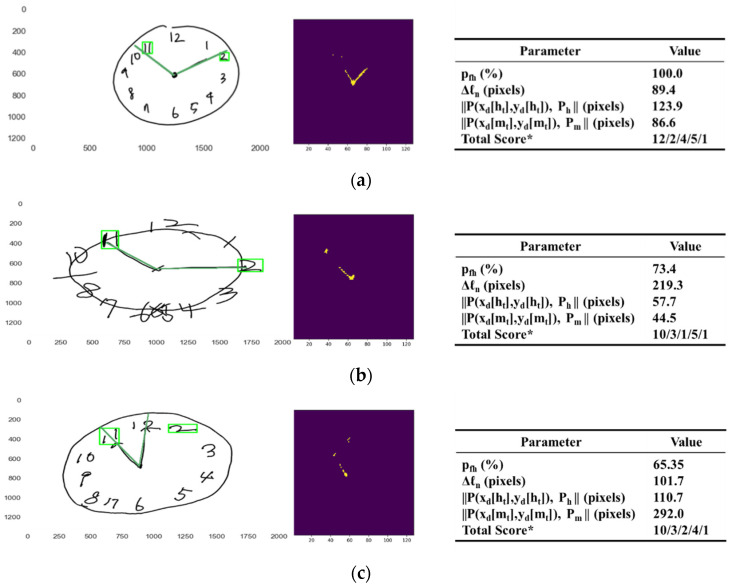

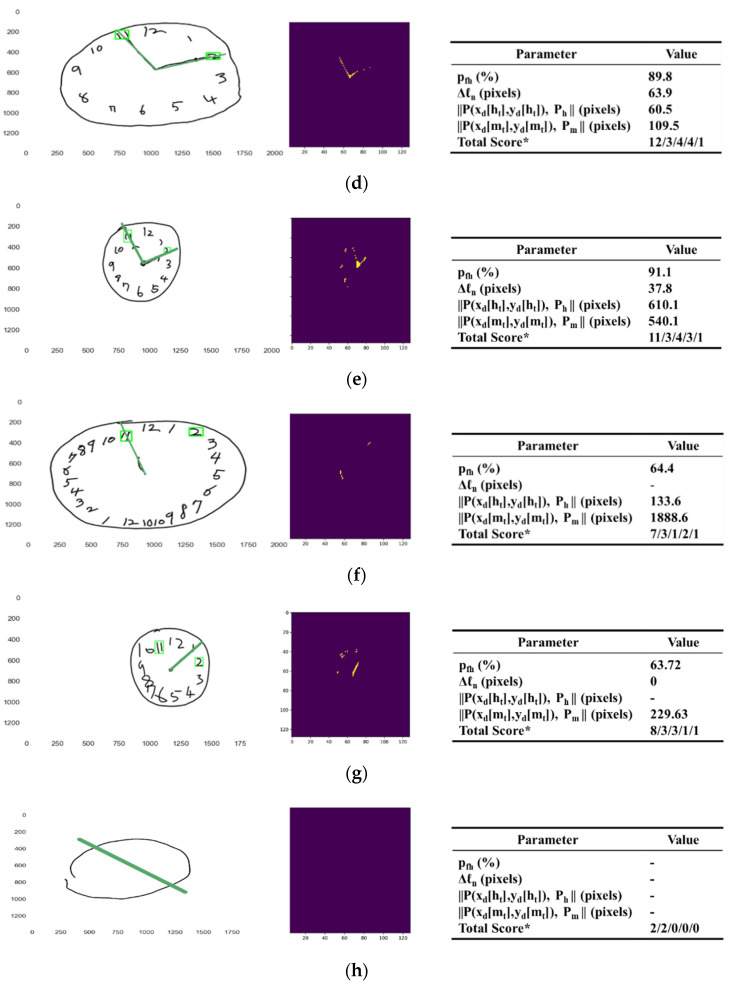
Eight representative examples demonstrating how numbers in original images are detected and classified in their orders and positions, showing the binarized image (**left**), the original image with the cropped areas for the numbers (**middle**), and the corresponding parameter values (**right**) of each examples; (**a**) the case in which two hands are present with the proper proportions and target numbers; (**b**) another example of the case in which two hands are present with the proper proportions and target numbers, where one of the target numbers is not in the proper position; (**c**) the case in which two hands are present with the proper proportions but one of them is not indicating the target number; (**d**) the case in which two hands are present with the proper target numbers but not the proper proportions; (**e**) the case in which two hands are present with the proper proportions but not the proper target numbers; (**f**) the case in which only one hand is present with the proper target number; (**g**) the case in which only one hand is present with neither the proper proportions nor the target numbers; and (**h**) the case in which no hands are present. * Total score/score of contour/score of numbers/score of hands/score of center.

The analytical ability of the pre-trained model DeepH is demonstrated in Figure 9, with eight separate examples on how two hands in the original images are evaluated in the presence, the correctness of the proportions, and the correctness of the target numbers, showing the segmented image of hands (left), the original image with the cropped areas of the target numbers, and the extrapolated lines of hands (middle), and the corresponding parameter values (right) of each examples.

Figure 9a displays the case in which two hands are present with the proper proportions and target numbers. In this case, the percentage $p_{fh}$ was evaluated to be 100.00% greater than 65% the score for $\theta_{h1}$. Therefore, the presence of two hands was scored to be two. The length difference $\Delta \ell_{h}$ was evaluated to be 89.4 pixels greater than 30.0 pixels the score for $\theta_{pr}$. Therefore, the correct proportion of the two hands was also scored to be one. Both of the distances $abs(P(x[n_{h}],y[n_{h}])-P(x[h_{t}],y[h_{t}]))$ and $abs(P(x[n_{m}],y[n_{m}])-P(x[m_{t}],y[m_{t}]))$ were estimated to be 123.9 and 86.6 pixels less than 200.0 pixels, the score for ε, respectively. Therefore, both the correctness of hand and minute target numbers were evaluated to be one. Finally, the total score of the hands parameter was evaluated to be five.

Figure 9b displays another example of the case in which two hands are present with the proper proportions and target numbers, where one of the target numbers is not in the proper position. In this case, the percentage $p_{fh}$ was evaluated to be 73.37% greater than 65% the score for $\theta_{h1}$. Therefore, the presence of two hands was scored to be two. The length difference $\Delta \ell_{h}$ was evaluated to be 219.3 pixels greater than 30.0 pixels, the score for $\theta_{pr}$. Therefore, the correct proportion of the two hands was also scored to be one. Both of the distances $abs(P(x[n_{h}],y[n_{h}])-P(x[h_{t}],y[h_{t}]))$ and $abs(P(x[n_{m}],y[n_{m}])-P(x[m_{t}],y[m_{t}]))$ were estimated to be 57.7 and 44.5 pixels less than 200.0 pixels, the score for ε, respectively. Therefore, both the correctness of hand and minute target numbers were evaluated to be one. Finally, the total score of the hands parameter was evaluated to be five.

Figure 9c displays the case in which two hands are present with the proper proportions, but one of them is not indicating the target number. In this case, the percentage $p_{fh}$ was evaluated to be 65.35% greater than 65%, the score for $\theta_{h1}$. Therefore, the presence of two hands was scored to be two. The length difference $\Delta \ell_{h}$ was evaluated to be 101.7 pixels less than 30.0 pixels, the score for $\theta_{pr}$. Therefore, the correct proportion of the two hands was also scored to be one. The distance $abs(P(x[n_{h}],y[n_{h}])-P(x[h_{t}],y[h_{t}]))$ was estimated to be 110.7 pixels less than 200.0 pixels, the score for ε, but the distance $abs(P(x[n_{m}],y[n_{m}])-P(x[m_{t}],y[m_{t}]))$ was estimated to be 292.0 pixels greater than 200.0 pixels, the score for ε. Therefore, the correctness of the hand target number was evaluated to be one, but the correctness of the minute target number was also evaluated to be one. Finally, the total score of the hands parameter was evaluated to be four.

Figure 9d displays the case in which two hands are present with the proper target numbers but not the proper proportions. In this case, the percentage $p_{fh}$ was evaluated to be 89.8% greater than 65.0%, the score for $\theta_{h1}$. Therefore, the presence of two hands was scored to be two. The length difference $\Delta \ell_{h}$ was evaluated to be 63.9 pixels less than 30.0 pixels, the score for $\theta_{pr}$. Therefore, the correct proportion of the two hands was also scored to be zero. Both of the distances $abs(P(x[n_{h}],y[n_{h}])-P(x[h_{t}],y[h_{t}]))$ and $abs(P(x[n_{m}],y[n_{m}])-P(x[m_{t}],y[m_{t}]))$ were estimated to be 60.5 and 109.5 pixels less than 200.0 pixels, the score for ε, respectively. Therefore, both the correctness of the hand and minute target numbers were evaluated to be one. Finally, the total score of the hands parameter was evaluated to be four.

Figure 9e displays the case in which two hands are present with the proper proportions but not the proper target numbers. In this case, the percentage $p_{fh}$ was evaluated to be 91.1% greater than 65.0%, the score for $\theta_{h1}$. Therefore, the presence of two hands was scored to be two. The length difference $\Delta \ell_{h}$ was evaluated to be 37.80 pixels greater than 30.0 pixels, the score for $\theta_{pr}$. Therefore, the correct proportion of the two hands was also scored to be one. Both of the distances $abs(P(x[n_{h}],y[n_{h}])-P(x[h_{t}],y[h_{t}]))$ and $abs(P(x[n_{m}],y[n_{m}])-P(x[m_{t}],y[m_{t}]))$ were estimated to be 610.1 and 540.1 pixels greater than 200.0 pixels, the score for ε, respectively. Therefore, both the correctness of hand and minute target numbers were evaluated to be zero. Finally, the total score of the hands parameter was evaluated to be three.

Figure 9f displays the case in which only one hand is present with the proper target number. In this case, the percentage $p_{fh}$ was evaluated to be 64.4% less than 65.0%, the score for $\theta_{h1}$, and greater than 50.0%, the score for $\theta_{h2}$. Therefore, the presence of two hands was scored to be one. The distance $abs(P(x[n_{h}],y[n_{h}])-P(x[h_{t}],y[h_{t}]))$ was estimated to be 133.6 pixels less than 200.0 pixels, the score for ε but the distance $abs(P(x[n_{m}],y[n_{m}])-P(x[m_{t}],y[m_{t}]))$ was estimated to be 1888.6 pixels greater than 200.0 pixels, the score for ε. Therefore, the correctness of the hand target number was evaluated to be one, and the correctness of the minute target number was also evaluated to be one. Finally, the total score of the hands parameter was evaluated to be two.

Figure 9g displays the case in which only one hand is present with neither the proper proportions nor the target numbers. In this case, the percentage $p_{fh}$ was evaluated to be 63.7% less than 65.0%, the score for $\theta_{h1}$ and greater than 50.0%, the score for $\theta_{h2}$. Therefore, the presence of two hands was scored to be one. Both of the distances $abs(P(x[n_{h}],y[n_{h}])-P(x[h_{t}],y[h_{t}]))$ and $abs(P(x[n_{m}],y[n_{m}])-P(x[m_{t}],y[m_{t}]))$ were estimated to be 229.63 greater than 200.0 pixels, the score for ε. Therefore, the correctness of hand target number was evaluated to be zero. Finally, the total score of the hands parameter was evaluated to be one.

Figure 9h displays the case in which no hands are present. Therefore, the total score of the hands parameter was evaluated to be zero.

### 3.4. Scoring on Criteria of Center Parameter

**Figure 10:**
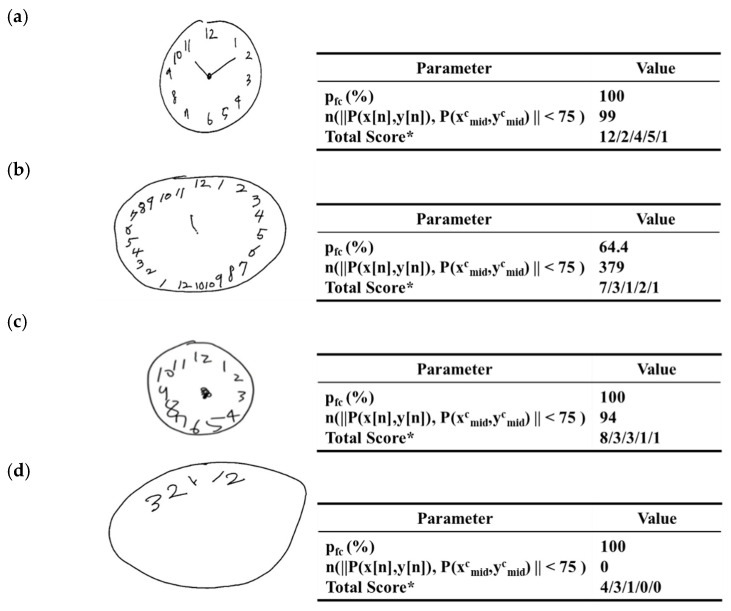
Four representative examples demonstrating how the centers in original images are detected to be present or inferred, showing the original image (**left**) and the corresponding parameter values (**right**) of each example; (**a**) the case in which two hands are present so the center is inferred; (**b**) the case in which only one hand is present so the center is inferred; (**c**) the case in which no hands are present but a data point exists near to the center of the contour; and (**d**) the case in which no center point is present or inferred. * Total score/score of contour/score of numbers/score of hands/score of center.

For presence or inference of a center in the hand drawn images, Figure 10 represents four cases of three having center points detected or inferred and the other having no center point. In each of the cases, the original clock drawing image example (left) and the corresponding parameter values (right) are presented.

Figure 10a displays the case in which two hands are present so the center is inferred. In this case, the percentage $p_{fh}$ was evaluated to be 100.00% greater than 65.0%, the score for $\theta_{h1}$. Therefore, the presence of a center point was evaluated to be one.

Figure 10b displays the case in which only one hand is present so the center is inferred. In this case, the percentage $p_{fh}$ was evaluated to be 64.4% less than 65.0%, the score for $\theta_{h1}$, and greater than 50.0%, the score for $\theta_{h2}$. This case also was evaluated to be one for the presence of a center point.

Figure 10c displays the case in which no hands are present, but a data point exists near to the center of the contour. Here, the number of the sensor data points P(x[n],y[n]) with distance $abs(P(x[n],y[n])-P(x_{mid}^{c},y_{mid}^{c}))$, less than 75.0 pixels, the given heuristic value of $\varepsilon_{c}$, from the predefined center point $P(x_{mid}^{c},y_{mid}^{c})$, which was evaluated to be 94. The presence of a center point was inferred and therefore evaluated to be one.

Figure 10d shows the case in which no center point is present or inferred. In this case, there are no hands and the number of the sensor data points P(x[n],y[n]) with distance $abs(P(x[n],y[n])-P(x_{mid}^{c},y_{mid}^{c}))$ less than 75.0 pixels, the given heuristic value of $\varepsilon_{c}$, from the predefined center point $P(x_{mid}^{c},y_{mid}^{c})$, which was evaluated to be zero. Therefore, the presence or the inference of a center was evaluated to be zero.

### 3.5. Performance Test Result

A total of 219 drawing images were used to test the performance of the scoring method by mCDT. Table 5 summarizes the frequency of the ground truth for the 219 images with the score in each of the parameters. For the parameter contour, the frequencies were 217, 178, and 215, with an error in estimation of 6, 13, and 1, respectively for the criteria of the circular contour, closure contour, and appropriately sized contour. For the parameter numbers, the frequencies were 153, 181, 88, and 202 with an error in estimation of 11, 5, 2, and 2, respectively, for the criteria of all the numbers present without additional numbers, numbers in corrected order, numbers in correct positions, and numbers within the contour. For the parameter hands, the frequencies were 171, 181, 170, 153, and 149 with an error in estimation of 13, 6, 13, 1, and 6, respectively, for the criteria of presence of two hands, presence of one hand, correct proportion of two hands, hour target number indication and minute target number indication. For the parameter center, the frequency was 190 with an error in estimation of three for the criteria of presence or inference of a center.

Table 6 and Table 7 list the distribution of the estimated scores and the performance of each scoring parameter, respectively in total as well as in the two separate groups, young volunteers and PD patients. As shown in Table 7, for the parameter contour, sensitivity, specificity, accuracy and precision, values were 89.33%, 92.68%, 89.95% and 98.15%; for numbers, they were 80.21%, 95.93%, 89.04% and 93.90%; for hands, they were 83.87%, 95.31%, 87.21% and 97.74%; and for center, they were 98.42%, 86.21%, 96.80% and 97.91%, respectively.

## 4. Discussion

A conventional CDT based on a paper and pencil test for examining the active and dynamic mechanisms of the cognitive function is inadequate. With the conventional CDT, multiple studies have indicated that a number of brain regions are recruited for the tasks required; these include the temporal lobes, frontal and parietal lobes in addition to the cerebellum, thalamus, premotor area and inferior temporal sulcus, the bilateral parietal lobe, and the sensorimotor cortex [39,40]. What is not clearly known are which portions of the cognitive function are required for recruiting these areas, as with the conventional CDT such an association and a quantitation would be difficult to accomplish. Our study sought to address this requirement from the CDT, and by introducing mCDT as a mobile phone application with a qualitative, automatic scoring system of CDT, this may have been realized. As elaborated previously, the mCDT scoring system was constructed using CNN, a convolutional network for digit classification, U-Net, a convolutional network for biomedical image segmentation, and the MNIST database, the Modified National Institute of Standards and Technology database. The sensor data is also collected by mCDT. From the performance test results, the scoring algorithm in mCDT is efficient and accurate when compared with those of the traditional CDT. In addition, mCDT is able to evaluate the relevant components of the cognitive function. The subjects in our study carried out the drawings with a smart pen on a smartphone screen when required to reproduce figures in a setting similar to the conventional CDT using a pen and paper. This method also allows for increased accuracy in gauging the drawing process and also minimizing any noise in an assay for activated brain function. The smartphone could also provide the motor-related markers of speed and pausing as the test is being carried out; in a conventional CDT pencil and paper test, such motor ability function cognitive tools may not be easily implemented. In summary, our study introduces the developed mCDT as a tool for increasing the accuracy required for the cognitive function evaluation in CDT. As described in the performance test results, mCDT showed fairly good statistical indicators, especially excellent values in specificity and precision. Furthermore, the values of specificity and precision for PD patient groups were better than those for the young volunteer group, which suggested that mCDT does classify the two groups very well and consistently so that it is applicable as a diagnostic tool in neurological disease group and also as a correlation tool between the scores of each criteria and the regional functions of the degenerated brain. Of course, the ability as a correlation tool needs to be investigated in future work, some preliminary studies of which is ongoing, with several clinical groups in collaboration with primary care physicians and neurology subspecialists. Furthermore, since the presented CDT scoring method here uses sensor data collected from a smart mobile device and deep learning based algorithm in the CDT image segmentation and processing, other stroke behavior patterns due to neurological disease symptoms such as motor, memory, and cognitive disorders could be additionally extracted using stroke speed variation and touch event sequence patterns that could be estimated from the sensor data; even the CDT scoring is limited to four parameters with thirteen criteria.

## 5. Conclusions

In this study, a mobile phone application mCDT for the CDT was implemented, and also an automatic and qualitative scoring method in thirteen criteria was developed using mobile sensor data and deep learning algorithms, U-Net and CNN. A young healthy volunteer (n = 238, 147 males and 89 females, aged 23.98 ± 2.83 years) and a PD patient (n = 140, 76 males and 64 females, aged 75.09 ± 8.57 years) group were recruited and participated in the training models DeepC, DeepH and DeepN, and in validating the performance of the CDT scoring algorithm. Most of the resulting overall statistical indicators, sensitivity, specificity, accuracy, and precision greater than 85%, were acquired at the performance validation of the 79 young healthy volunteers and 140 PD patients. Two exceptions were recognized at the sensitivities of the number and the hands parameters. Especially, the specificities of the contour and hand parameter of the young volunteer group were shown to be far too low (60.00% and 66.67%, respectively), which was because the number of true negatives and false positives were a lot smaller, as well as they were in relatively similar proportions. Furthermore, the specificities and the precisions of the PD patients group were better than those of the young volunteer group, which suggests that the mCDT along with the scoring method is available to be used as a tool of classifying neurological disease groups and also as a tool of scaling the disease symptoms related to degenerated regions of the brain. Further clinical studies should be established in differentiating neurological disease subtypes, being valuable in clinical practice and for studies in the field.

## Figures and Tables

**Table 1:** Statistics of age, gender, handedness and clinical status of the participants.

	Training Set	Test Set	
	Volunteers (*n* = 159)	Volunteers (*n* = 79)	PD Patients (*n* = 140)
Age	24.78 ± 1.63	22.81 ± 0.79	75.09 ± 8.57
Male (female)	112 (45)	35 (44)	76 (64)
Binary CDT score Pass (Non-pass)	159 (0)	75 (4)	73 (67)

**Table 2:** Detailed list of the parameters for the scoring method.

Parameters	Scoring Criteria
**Contour**	Contour is circular
	Contour is closed
	Contour size is appropriate
**Numbers**	Numbers are all present without additional numbers
	Numbers are in the corrected order
	Numbers are in the correct positions
	Numbers are within the contour
**Hands**	Two hands are present
	One hand is present
	Hour target number is indicated
	Minute target number is indicated
	Hands are in correct proportion
**Center**	A center is drawn or inferred
**Total**	0–13 scores

**Table 3:** Formulas of the pre-estimated positions of the number digits from 0 to 12.

Number Digit k	Formula	
	$x_{d}[k]$	$y_{d}[k]$
1	$(2x_{mid}^{c}+x_{\max}^{c})/3$	$(2y_{\max}^{c}+y_{mid}^{c})/3$
2	$(x_{mid}^{c}+2x_{\max}^{c})/3$	$(y_{\max}^{c}+2y_{mid}^{c})/3$
3	$x_{\max}^{c}$	$y_{mid}^{c}$
4	$(x_{mid}^{c}+2x_{\max}^{c})/3$	$(y_{\min}^{c}+2y_{mid}^{c})/3$
5	$(2x_{mid}^{c}+x_{\max}^{c})/3$	$(2y_{\min}^{c}+y_{mid}^{c})/3$
6	$x_{mid}^{c}$	$y_{\min}^{c}$
7	$(2x_{mid}^{c}+x_{\min}^{c})/3$	$(2y_{\min}^{c}+y_{mid}^{c})/3$
8	$(x_{mid}^{c}+2x_{\min}^{c})/3$	$(y_{\min}^{c}+2y_{mid}^{c})/3$
9	$x_{\min}^{c}$	$y_{mid}^{c}$
10	$(x_{mid}^{c}+2x_{\min}^{c})/3$	$(y_{\max}^{c}+2y_{mid}^{c})/3$
11	$(2x_{mid}^{c}+x_{\min}^{c})/3$	$(2y_{\max}^{c}+y_{mid}^{c})/3$
12	$x_{mid}^{c}$	$y_{\max}^{c}$

**Table 4:** Details for assignment of scores.

**Parameters**	**Criteria**	Conditions (Scoring Method) *
Contour	circular contour	$p_{fc}\geq \theta_{c1}$ ^1^
	closed contour	$d_{\max}^{c}\geq \theta_{c2}$ ^2^
	appropriately sized contour	$A_{c}/W_{c}\geq \theta_{c3}$ ^3^
Numbers	all and no additional numbers	N=15 & 0≤D[j]≤9 for all *j* & *n*(*D*[*j*] = 1) = 5 & *n*(*D*[*j*] = 2) = 2
	correct order of numbers	$R_{seq}\geq \theta_{n}$ ^4^
	correct position of numbers	$n(d_{cn}[j]\leq \ell_{dc}{}^{5})/n(d_{cn}[j])>\theta_{dc}{}^{6}$
	positioning of numbers within contour	$R_{cN}<R_{cL}$,
Hands	two hands	$p_{fh}>\theta_{h1}$ ^7^
	one hand	$p_{fh}>\theta_{h2}$ ^8^
	correct proportion of hands	$p_{fh}>\theta_{h1}$ & $abs(n(S_{1})-n(S_{2}))>\theta_{pr}$ ^9^
	correct hour target number	$abs(P(x[n_{h}],y[n_{h}])-P(x[h_{t}],y[h_{t}]))<\varepsilon$ ^10^
	correct minute target number	$abs(P(x[n_{m}],y[n_{m}])-P(x[m_{t}],y[m_{t}]))<\varepsilon$
Center	existence or inference of a center	$p_{fh}>\theta_{h1}$ or $abs(P(x[n],y[n])-P(x_{mid}^{c},y_{mid}^{c}))<\varepsilon_{c}$ ^11^
Total sum	13	

* heuristic values of the thresholds: ^1^ $\theta_{c1}=75.00$ pixels; ^2^ $\theta_{c2}=50.00$ pixels; ^3^ $\theta_{c3}=0.1$; ^4^ $\theta_{n}=65.00$ pixels; ^5^ $\ell_{dc}=100.00$ pixels; ^6^ $\theta_{dc}=0.65$; ^7^ $\theta_{h1}=65.00$ pixels; ^8^ $\theta_{h2}=50.00$ pixels; ^9^ $\theta_{pr}=30.00$ pixels; ^10^ ε=200.00 pixels; ^11^ $\varepsilon_{c}=75.00$ pixels.

**Table 5:** Frequency of the ground truth of the 219 images in each criteria of the parameters of the scoring method of mCDT.

Parameter	Criteria	Frequency Count (%)	Errors in Estimation Count (%)
**Contour**	Contour is circular	217(99.08)	6(2.76)
	Contour is closed	178(81.27)	13(7.30)
	Contour size is appropriate	215(98.17)	1(0.46)
**Numbers**	Numbers are all present without additional numbers	153(69.86)	11(7.18)
	Numbers are in corrected order	181(82.64)	5(2.76)
	Numbers are in the correct positions	88(40.18)	2(2.27)
	Numbers are within the contour	202(92.23)	2(0.99)
**Hands**	Two hands are present	171(78.08)	13(7.60)
	One hand is present	181(82.64)	6(3.31)
	Hands are in correct proportion	170(77.62)	13(7.64)
	Hour target number is indicated	153(69.86)	1(0.65)
	Minute target number is indicated	149(68.03)	6(4.02)
**Center**	A center is drawn or inferred	190(86.75)	3(1.57)

**Table 6:** Distribution of the estimated scores for each scoring parameters in mCDT.

Scores	Contour	Numbers	Hands	Center
**5**	-	-	144 (70/74)	-
**4**	-	81 (53/28)	11 (3/8)	-
**3**	175 (74/101)	71 (23/48)	15 (6/9)	-
**2**	42 (5/37)	30 (3/27)	4 (0/4)	-
**1**	1 (0/1)	27 (0/27)	7 (0/7)	190 (79/111)
**0**	1 (0/1)	10 (0/10)	38 (0/38)	29 (0/29)
**Total**	219 (79/140)	219 (79/140)	219 (79/140)	219 (79/140)

**Table 7:** Performance of the scoring parameters in mCDT.

	Contour	Numbers	Hands	Center
**TP**	159 (70/89)	77 (50/27)	130 (66/64)	187 (79/108)
**FP**	3 (2/1)	5 (4/1)	3 (3/0)	4 (0/4)
**FN**	19 (4/15)	19 (5/14)	25 (4/21)	3 (0/3)
**TN**	38 (3/35)	118 (20/98)	61 (6/55)	25 (0/25)
**Sensitivity**	89.33 (94.60/85.58)	80.21 (90.91/65.85)	83.87 (94.29/75.29)	98.42 (100.00/97.30)
**Specificity**	92.68 (60.00/97.22)	95.93 (83.33/98.99)	95.31 (66.67/100.00)	86.21 (-/86.21)
**Accuracy**	89.95 (92.41/88.57)	89.04 (88.61/89.29)	87.21 (91.14/85.00)	96.80 (100.00/95.00)
**Precision**	98.15 (97.22/98.89)	93.90 (92.59/96.43)	97.74 (95.65/100.00)	97.91 (100.00/96.43)

## Data Availability

The data presented in this study are available on request from the corresponding author. The data are not publicly available due to privacy issue.
